# Natural Language Processing and Machine Learning Methods to Characterize Unstructured Patient-Reported Outcomes: Validation Study

**DOI:** 10.2196/26777

**Published:** 2021-11-03

**Authors:** Zhaohua Lu, Jin-ah Sim, Jade X Wang, Christopher B Forrest, Kevin R Krull, Deokumar Srivastava, Melissa M Hudson, Leslie L Robison, Justin N Baker, I-Chan Huang

**Affiliations:** 1 Department of Biostatistics St. Jude Children's Research Hospital Memphis, TN United States; 2 Department of Epidemiology and Cancer Control St. Jude Children's Research Hospital Memphis, TN United States; 3 School of AI Convergence Hallym University Chuncheon Republic of Korea; 4 Roberts Center for Pediatric Research Children's Hospital of Philadelphia Philadelphia, PA United States; 5 Department of Oncology St. Jude Children's Research Hospital Memphis, TN United States

**Keywords:** natural language processing, machine learning, PROs, pediatric oncology

## Abstract

**Background:**

Assessing patient-reported outcomes (PROs) through interviews or conversations during clinical encounters provides insightful information about survivorship.

**Objective:**

This study aims to test the validity of natural language processing (NLP) and machine learning (ML) algorithms in identifying different attributes of pain interference and fatigue symptoms experienced by child and adolescent survivors of cancer versus the judgment by PRO content experts as the gold standard to validate NLP/ML algorithms.

**Methods:**

This cross-sectional study focused on child and adolescent survivors of cancer, aged 8 to 17 years, and caregivers, from whom 391 meaning units in the pain interference domain and 423 in the fatigue domain were generated for analyses. Data were collected from the After Completion of Therapy Clinic at St. Jude Children’s Research Hospital. Experienced pain interference and fatigue symptoms were reported through in-depth interviews. After verbatim transcription, analyzable sentences (ie, meaning units) were semantically labeled by 2 content experts for each attribute (physical, cognitive, social, or unclassified). Two NLP/ML methods were used to extract and validate the semantic features: bidirectional encoder representations from transformers (BERT) and Word2vec plus one of the ML methods, the support vector machine or extreme gradient boosting. Receiver operating characteristic and precision-recall curves were used to evaluate the accuracy and validity of the NLP/ML methods.

**Results:**

Compared with Word2vec/support vector machine and Word2vec/extreme gradient boosting, BERT demonstrated higher accuracy in both symptom domains, with 0.931 (95% CI 0.905-0.957) and 0.916 (95% CI 0.887-0.941) for problems with cognitive and social attributes on pain interference, respectively, and 0.929 (95% CI 0.903-0.953) and 0.917 (95% CI 0.891-0.943) for problems with cognitive and social attributes on fatigue, respectively. In addition, BERT yielded superior areas under the receiver operating characteristic curve for cognitive attributes on pain interference and fatigue domains (0.923, 95% CI 0.879-0.997; 0.948, 95% CI 0.922-0.979) and superior areas under the precision-recall curve for cognitive attributes on pain interference and fatigue domains (0.818, 95% CI 0.735-0.917; 0.855, 95% CI 0.791-0.930).

**Conclusions:**

The BERT method performed better than the other methods. As an alternative to using standard PRO surveys, collecting unstructured PROs via interviews or conversations during clinical encounters and applying NLP/ML methods can facilitate PRO assessment in child and adolescent cancer survivors.

## Introduction

### Pediatric Cancer and Patient-Reported Outcomes

Innovative anticancer therapies have significantly improved the 5-year survival rates of pediatric and adolescent patients with cancer in the United States [1-3]. However, toxic treatment often causes long-term sequelae (eg, physical and psychological morbidities and premature mortality [4-8]), which contribute to poor patient-reported outcomes (PROs) and impaired quality of life [8,9]. Poor PROs, such as fatigue, pain, psychological distress, and neurocognitive problems, are prevalent in survivors of cancer aged <18 years [10-12]. Approximately 50% of young survivors of childhood cancer experience severe fatigue [10,12,13] or pain [12,14], and both can worsen as survivors become older [15]. Assessing PROs from survivors and caregivers can complement clinical assessments, suggest potential adverse medical events, and facilitate the provision of appropriate interventions [16,17].

### Unstructured PROs

Conventionally, PROs are collected from childhood survivors of cancer during follow-up care using standard surveys with prespecified content of PROs. Given busy clinic schedules, survivors may be unable or unwilling to complete surveys. Performing interviews or initiating conversations by clinicians are alternative methods of collecting PROs. However, PROs collected by this method are qualitative or unstructured in nature, which requires specific techniques for data processing and analysis. Natural language processing (NLP), a discipline of linguistics, information engineering, and artificial intelligence, initially designed for processing a large amount of natural language data, provides an innovative avenue for PRO research with potential clinical applications [18]. However, the validity of applying this method to evaluate PROs in oncology is understudied.

### Application of NLP for PRO Analysis

NLP techniques have been applied to process unstructured or nonquantitative clinical data in medical notes for classifying or predicting health status (eg, risk of heart disease and stage of cancer) through information extraction, semantic representation learning, and outcome prediction [19]. Recently, NLP applications have been extended to unstructured PRO and symptom data stored in electronic medical records (EMRs) [20,21]. A review study [22] found that most previous NLP applications for unstructured PRO data largely focused on rule-based classifications (eg, extracting prespecified keywords or phrases from free text to identify cancer-related symptoms [23]), followed by machine learning (ML) approach (eg, conditional random field model [20], support vector machine [SVM] [24], and boosting regression tree [25]) to analyze associations with clinical outcomes.

The method of capturing the features of unstructured PROs is an emerging area of research [26]. Compared with rule-based extraction, the ML/deep learning–based NLP methods, including the context-independent or static (eg, term frequency–inverse document frequency [TF-IDF] [27], global vectors for word representation [GloVe] [28], and Word2vec [29]), and context-dependent or dynamic (eg, bidirectional encoder representations from transformers [BERT]; [30]) distributed representation methods are more suitable for processing unstructured PROs. Typically, context-dependent methods can capture the meaning of polysemous words, which substantially improves the flexibility and validity of analyzing unstructured PRO data.

### Objective

To facilitate clinical decisions, our long-term goal is to collect PROs from survivor-caregiver-clinician conversations and apply NLP/ML methods to characterize meaningful PROs. Through in-depth interviews with childhood survivors of cancer and caregivers, this study evaluates the validity of using different novel NLP/ML methods (Word2vec/ML and BERT) to characterize 2 most common symptom domains (pain interference and fatigue) in child and adolescent survivors of cancer. The interview data were semantically labeled and coded by PRO content experts as the gold standard to represent specific symptom problems (defined as symptom attributes). In contrast to the static methods (ie, Word2vec/ML), we hypothesize that the use of dynamic methods (ie, BERT) would yield superior model performance.

## Methods

### Study Participants

Study participants were survivors of pediatric cancer and their caregivers recruited from the After Completion of Therapy Clinic at St. Jude Children’s Research Hospital (*St Jude* hereafter) in Tennessee, United States, between August and December 2016. Eligible participants were identified from a list of survivors scheduled for annual follow-up and confirmed their eligibility through EMRs. We recruited survivors aged 8 to 17 years of age at annual follow-up, at least 2 years off therapy, and at least 5 years from initial cancer diagnosis. We excluded survivors who had acute or life-threatening conditions and required immediate medical care. We recruited caregivers who were the most knowledgeable of the survivor’s health status and could speak or read English. Assent from survivors and consent from caregivers was obtained. The research protocol was approved by the institutional review board of St Jude.

### In-Depth Interview and Data Abstraction

This investigation builds on our previous study that elucidated the contents of 5 PRO domains (pain interference, fatigue, psychological stress, stigma, and meaning and purpose) related to pediatric cancer from survivors and caregivers [15]. We randomly assigned 2 domains to each survivor and 2 to 3 domains to each caregiver. PRO domains were assigned randomly to each survivor and caregiver to elucidate PRO contents from both survivors and caregivers rather than comparing PRO discordances between dyadic participants. Diagnostic and clinical information was abstracted from EMRs. We designed separate interview guides (Multimedia Appendices 1 and 2) with probes for each PRO domain, audio-recorded the interviews, transcribed interviews verbatim, and abstracted meaningful and interpretable sentences (ie, “meaning units”) [15].

### Expert-Labeled Outcomes as the Gold Standard

We used the methods developed in our previous studies to code the concepts of symptomatic problems collected from interviews and assigned the concepts to specific attributes [15,31]. Specifically, we began with abstracting the sentences or paragraphs collected from the interviews that are relevant to the experiences with particular symptomatic problems, such as presence, frequency, or intensity, and how these symptomatic problems affect daily activities (defined as meaning units) and then mapped the meaning units to analyzable, interpretable formats that represent the contents of items included in the Patient-Reported Outcomes Measurement Information System (PROMIS) banks [32] (defined as meaningful concepts). Subsequently, we labeled the meaningful concepts by distinct concepts, including physical, cognitive, and social (defined as attributes) concepts.

The associations among meaning units, meaningful concepts, and corresponding attributes are illustrated in Multimedia Appendix 3. For example, in the pain interference domain, when a survivor stated that “Can’t play, and go outside when I have a headache,” we mapped this meaning unit to the meaningful concept “Hard to do sports or exercise when had pain,” and then labeled this meaningful concept as the *physical* attribute. For the fatigue domain, when a survivor stated that “It’s hard to get my school work done when I’m tired,” we mapped this meaning unit to the meaningful concept “Hard to keep up with schoolwork” and then labeled this meaningful concept as the *cognitive* attribute.

In addition, 2 PRO content experts (JLC and CMJ) independently reviewed the content of each meaning unit derived from the symptom domains and mapped each meaning unit to the content of individual items listed in the PROMIS pain interference and fatigue item banks [32]. In total, 391 and 423 meaning units representing pain interference and fatigue domains, respectively, were included in the analysis, and each meaning unit was labeled and coded as problematic symptoms based on key attributes (physical, cognitive, social, and unspecified). Discrepancies in the mapping process were resolved by consensus between 2 senior investigators (CBF and ICH). PROMIS has applied rigorous standards to develop a comprehensive list of PRO items, therefore serving as a foundation for evaluating PRO contents [33-37]. This mapping process has been adopted in previous research to facilitate the abstraction and mapping of qualitative data [38-40]. In this study, the expert-labeled symptoms attributed to each meaning unit were deemed the gold standard for testing the validity of NLP/ML methods.

We evaluated the interrater reliability based on the raw concordance rate (defined as the percentage of coded meaning units that 2 coders provide concordant ratings), and Cohen κ statistic (defined as the number of concordant ratings to the number of discordant ratings while considering the agreement that is expected by chance). In our study, raw concordance rates were 88% for the pain interference domain and 86% for the fatigue domain. Cohen κ statistic was 0.6 for both domains, which is considered moderate or good reliability for coding qualitative PRO data [41].

### NLP/ML Pipeline

**Figure 1:**
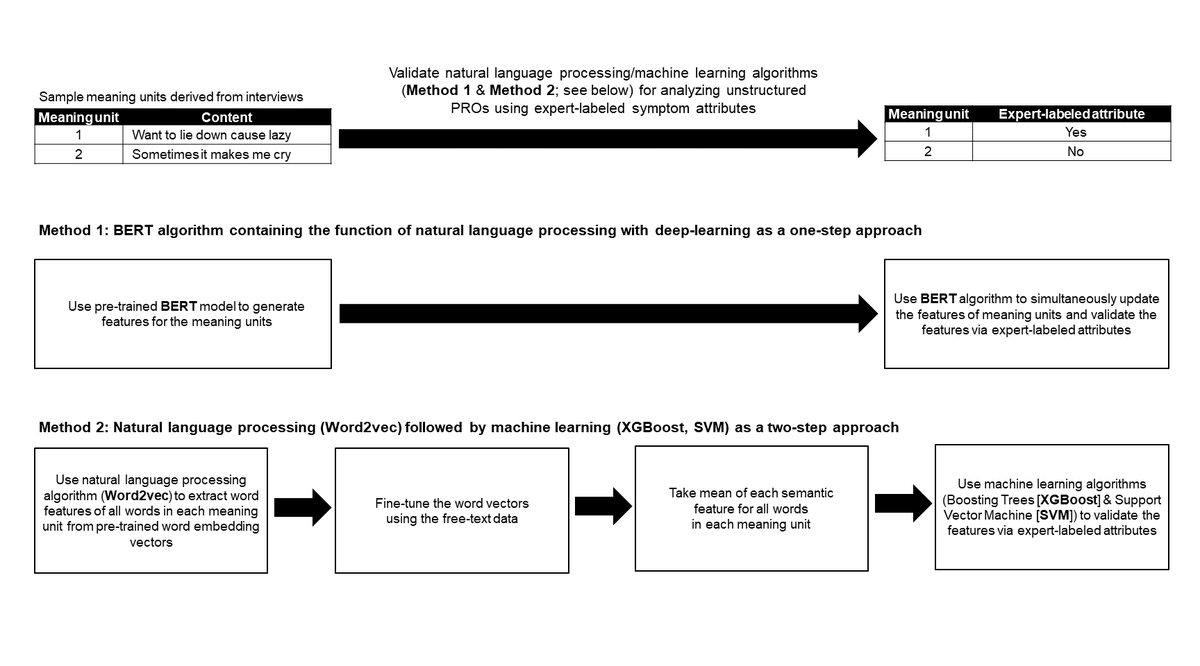
The natural language processing and machine learning pipeline to analyze unstructured patient-reported outcomes data. BERT: bidirectional encoder representations from transformers; PROs: patient-reported outcomes; SVM: support vector machine; XGBoost: extreme gradient boosting.

Figure 1 outlines the pipeline of NLP/ML methods consisting of 2 key components: (1) extracting semantic features from the unstructured PROs and (2) using expert-labeled attributes of symptoms to validate NLP/ML–generated semantic features. We used the Word2vec [29] and BERT [30] methods to create multivariate semantic features (ie, word vectors) for each word from the meaning units. The BERT method embeds deep neural networks as a single step to perform abstraction and validation for the semantic features of symptom data simultaneously, whereas Word2vec/ML techniques involve 2 separate steps to achieve these tasks (Figure 1 and Multimedia Appendix 4).

### BERT (Base, Uncased) for PRO Feature Extraction and Validation

The BERT (base, uncased; or the *BERT* hereafter), our primary interest in the NLP method, consists of the multilayer neural networks known as encoder transformers, and each generates context-dependent word features by weighting the features of each word with the other words in the meaning units [30,42]. We used 12 stacked layers of encoders to explore phrase-level, syntactic, semantic, and contextual information [42]. Specifically, we used the semantic features pretrained by articles published in BooksCorpus and Wikipedia to generate general word semantic meanings (pretrained model in Multimedia Appendix 5 [30,43]). The BERT model is augmented with a classification component, consisting of a feed-forward neural network and a softmax layer [44] to classify unstructured PROs (fine-tuning process in Multimedia Appendices 5 and 6). This augmented model was fine-tuned by the meaning units collected from interviews, which adapts the sentence contextual representation in encoders to the symptom-related contexts, and the parameters in the classification component were estimated simultaneously in one step.

Specifically, we used the pretrained model (BERT [base, uncased]) from the huggingface model repository, which was a pytorch implementation of the base BERT model [30]. The pretrained model is essentially based on the text passages included in BooksCorpus [43] and the English Wikipedia [30]. The weight parameters in the pretrained BERT model were further fine-tuned with the texts in the meaning units from our interview data when the BERT model was used for the downstream classification task of the meaning units through the BertForSequenceClassification object in the pytorch_transformers module. The use of BooksCorpus and Wikipedia is appropriate for our survivors of pediatric cancer as both contain comprehensive generic terms that capture the heterogeneous health status experienced by varying survivors of cancer, ranging from healthy (no late effects and no symptoms) to ill (severe late effects with severe symptoms).

### Word2vec Method for PRO Feature Extraction and ML for Validation

We used Word2vec, our secondary interest in the NLP method, to extract semantic features based on the similarity of words in meaning units. Embedded with a one-level neural network model (Multimedia Appendix 7), Word2vec defines the semantic similarity across different words by using a specific word to search and connect other words nearby, given the hypothesis that a word’s meaning is given by adjacent words [45,46]. We adopted the semantic features already pretrained by English articles from Wikipedia [47,48] to generate and fine-tune the semantic meanings of the meaning units through our data (Figure 1; Multimedia Appendices 6 and 7).

We used 2 ML methods, including the extreme gradient boosting (XGBoost) [25] and the SVM [24], to validate the semantic features derived from Word2vec in associations with the expert-labeled symptom attributes. ML modeling was used to account for high dimensional structures of semantic features created by Word2vec [29] (Multimedia Appendix 7). Specifically, XGBoost is a robust regression tree approach that includes multiple simple decision trees to iteratively refine the model performance by minimizing the difference between the expected and expert-labeled outcomes. In contrast, SVM is a classical ML algorithm that aims to find a decision boundary to separate the semantic features corresponding to the expert-labeled attributes by minimizing classification errors.

### Alternative Methods for PRO Feature Extraction

In addition to the BERT, Word2vec/SVM, and Word2vec/XGBoost models, we conducted pilot analyses to evaluate 6 alternative NLP/ML models, including the TF-IDF/SVM, GloVe/SVM, and GloVe/XGBoost, as well as 3 extended BERT models (BioBERT, BlueBERT, and Clinical BERT). Briefly, the TF-IDF is an automatic text analysis that accounts for the number of times a word appears in a document and the number of documents that contain the word [27]. The GloVe method identifies the global word similarity over several meaning units (ie, our unit of analysis) or the entire interview [28]. The 3 alternative BERT models for pilot testing included the BioBERT (base, cased and trained on PubMed 1M) [49], BlueBERT (base, uncased and trained on PubMed) [50], and Clinical BERT (base, cased, initialized from BioBERT and trained on all MIMIC-III notes) [51].

As demonstrated in Multimedia Appendix 8, the areas under the precision-recall (PR) curves for the BERT model were significantly superior to the TF-IDF/SVM, GloVe/SVM, and GloVe/XGBoost (all attributes over 2 symptom domains) and were significantly superior to the BioBERT, BlueBERT, and Clinical BERT models (especially physical and cognitive attributes in the pain interference domain). In addition, the use of GloVe/SVM, Word2vec/SVM, and Word2vec/XGBoost methods resulted in statistically nonsignificant differences. Model performances based on other evaluation metrics were reported in Multimedia Appendices 9 and 10. As the main purpose of this study was to identify the NLP/ML model with optimal performance for symptom assessment, we focused on comparisons between the BERT model (as a theoretically optimal method) and the Word2vec model accompanied by SVM and XGBoost (as a suboptimal method).

### Model Training and Evaluation

We used a 5-folder nested cross-validation approach (Multimedia Appendix 11) to address the issue of small sample size, including the components of partitioning the training, validation and test sets, determining the tuning parameters in ML methods, and generating validation results. Given the 4-attribute classification (physical, cognitive, social, and unclassified) on each meaning unit, we used a one-versus-rest binary classifier to classify one attribute (physical, cognitive, or social) versus the remaining attributes (the reference) for model training and evaluation [52].

We used standard metrics to test the validity of NLP/ML models, including precision (ie, positive predictive value), sensitivity (ie, recall), specificity, accuracy (summarizing true positive and true negative), F1 score (summarizing sensitivity and positive predictive values), areas under the receiver operating characteristic (ROC) curve, and areas under the PR curve. In the case of imbalanced data (ie, a limited number of meaning units labeled as attribute presence versus that of the reference), the PR curve is more suitable than the ROC curve as the former focuses on precision and sensitivity related to true positive cases [53]. On the basis of a recommendation [53], we determined the baseline threshold for each attribute of a symptom domain as the percentage of meaning units that were rated by 2 coders or content experts (ie, the gold standard for labeling true presence of attribute), which represents the precision of a random guess classifier.

Our NLP framework benefits from the transfer learning framework, which uses a huge amount of related data in the public domains to improve the ML application with regular sample sizes. Specifically, our Word2vec and BERT models or algorithms were pretrained by millions of health-related information in the public domains (eg, Wikipedia). Our meaning units were only used to fine-tune or improve the pretrained model and as predictive samples. Although our sample size was not large, it was sufficient to achieve robust validation and predictive performance. The codes used for BERT modeling are available on the GitHub website [54]; the fully deidentified unstructured PRO data used in this study can be shared for research purposes on user’s request.

## Results

### Participant Characteristics

Table 1 reports the participant characteristics. The mean (SD) ages of survivors (N=52) and caregivers (N=35) at interviews were 13.8 (2.8) and 39.6 (7.0) years, respectively. Approximately 42% (22/52) of survivors were treated for noncentral nervous system solid tumors and 33% (17/52) for leukemia. For meaning units, 391 in the pain interference domain—of the 391 units, 255 (65.2%) were from survivors, and 136 (34.8%) were from caregivers—and 423 in the fatigue domain—of the 423 units, 275 (65%) were from survivors, and 148 (35%) were from caregivers— were labeled and analyzed accordingly (Multimedia Appendix 12).

**Table 1:** Characteristics of study participants (N=87).

Characteristics			Survivors (n=52)		Caregivers (n=35)
Age at evaluation (years), mean (SD)			13.8 (2.8)		39.6 (7.0)
**Sex, n (%)**					
	Female	31 (61)		32 (91)	
	Male	20 (39)		3 (9)	
**Race or ethnicity, n (%)**					
	White, non-Hispanic	30 (59)		24 (69)	
	Black, non-Hispanic	14 (28)		10 (29)	
	Other	7 (14)		1 (3.0)	
**Cancer diagnosis, n (%)**					
	Non-CNS^a^ solid tumor	22 (42)		N/A^b^	
	Leukemia	17 (33)		N/A	
	CNS malignancy	9 (17)		N/A	
	Lymphoma	4 (8.0)		N/A	

^a^CNS: central nervous system.

^b^N/A: not applicable.

### Sensitivity, Specificity, Precision, and Accuracy for Pain Interference

Table 2 reports the model performance for the pain interference domain based on survivor and caregiver data. For the sensitivity metric, compared with Word2vec/SVM and Word2vec/XGBoost, BERT generated higher values in identifying problems with 3 attributes (physical, cognitive, and social); however, the values were largely <0.6. In contrast, all 3 methods produced specificity of >0.9, and Word2vec/XGBoost produced higher values in identifying problems with 3 attributes compared with BERT and Word2vec/SVM. For F1-statistics, BERT yielded higher values for all 3 attributes compared with Word2vec/SVM and Word2vec/XGBoost. BERT yielded higher accuracy for all 3 attributes compared with Word2vec/SVM and Word2vec/XGBoost; the values were all >0.8, specifically 0.931 (95% CI 0.905-0.957), 0.916 (95% CI 0.887-0.941), and 0.870 (95% CI 0.836-0.903) for cognitive, social, and physical attributes, respectively.

**Table 2:** Performance of natural language processing/machine learning models for pain interference domain by 3 symptom attributes.

Attributes and models		Precision (95% CI)	Sensitivity (95% CI)	Specificity (95% CI)	Accuracy (95% CI)	F1 (95% CI)	AUROCC^a^ (95% CI)	AUPRC^b^ (95% CI)
**Physical**								
	BERT^c^	0.692 (0.555-0.811)	0.507 (0.387-0.618)	0.950 (0.924-0.972)	0.870 (0.836-0.903)	0.585 (0.467-0.683)	0.875 (0.824-0.948)	0.677 (0.568-0.770)
	Word2vec/SVM^d^	0.722 (0.562-0.867)	0.366 (0.262-0.479)	0.969 (0.948-0.987)	0.859 (0.824-0.893)	0.486 (0.362-0.594)	0.868 (0.826-0.922)	0.623 (0.5090.743)
	Word2vec/XGBoost^e^	0.697 (0.528-0.857)	0.324 (0.221-0.435)	0.969 (0.949-0.987)	0.852 (0.813-0.887)	0.442 (0.318-0.551)	0.830 (0.769-0.888)	0.553 (0.437-0.659)
**Cognitive**								
	BERT	0.800 (0.657-0.935)	0.583 (0.432-0.735)	0.980 (0.964-0.994)	0.931 (0.905-0.957)	0.675 (0.543-0.779)	0.923 (0.879-0.997)	0.818 (0.735-0.917)
	Word2vec/SVM	0.760 (0.583-0.920)	0.396 (0.254-0.533)	0.983 (0.967-0.994)	0.910 (0.882-0.939)	0.521 (0.361-0.648)	0.900 (0.863-0.957)	0.609 (0.434-0.761)
	Word2vec/XGBoost	0.769 (0.500-1.000)	0.208 (0.104-0.333)	0.991 (0.980-1.000)	0.895 (0.867-0.926)	0.328 (0.178-0.474)	0.828 (0.748-0.905)	0.474 (0.321-0.630)
**Social**								
	BERT	0.636 (0.461-0.800)	0.500 (0.349-0.652)	0.966 (0.946-0.983)	0.916 (0.887-0.941)	0.560 (0.410-0.690)	0.857 (0.786-0.918)	0.566 (0.402-0.750)
	Word2vec/SVM	0.286 (0-0.668)	0.048 (0-0.118)	0.986 (0.973-0.997)	0.885 (0.854-0.916)	0.082 (0.035-0.200)	0.804 (0.742-0.878)	0.309 (0.173-0.426)
	Word2vec/XGBoost	0.556 (0.222-0.875)	0.119 (0.029-0.229)	0.989 (0.977-0.997)	0.895 (0.864-0.923)	0.196 (0.072-0.343)	0.786 (0.728-0.850)	0.304 (0.148-0.420)

^a^AUROCC: area under the receiver operating characteristic curve.

^b^AUPRC: area under precision-recall curve.

^c^BERT: bidirectional encoder representations from transformers.

^d^SVM: support vector machine.

^e^XGBoost: extreme gradient boosting.

### Sensitivity, Specificity, Precision, and Accuracy for Fatigue

Table 3 reports the model performance for the fatigue domain based on the survivor and caregiver data. For sensitivity, the BERT method generated higher values in identifying problems with 3 attributes compared with Word2vec/SVM and Word2vec/XGBoost; however, the values were largely <0.5, except cognitive attributes (0.757). In contrast, all 3 methods produced specificity >0.9, and Word2vec/SVM produced higher values in identifying problems with 3 attributes compared with BERT and Word2vec/XGBoost. The BERT model yielded higher F1-statistics for all 3 individual attributes compared with Word2vec/SVM and Word2vec/XGBoost. In addition, the BERT model produced higher accuracy for all 3 attributes compared with Word2vec/SVM and Word2vec/XGBoost; the values were all >0.8, specifically 0.929 (95% CI 0.903-0.953), 0.917 (95% CI 0.891-0.943), and 0.832 (95% CI 0.794-0.867) for cognitive, social, and physical attributes, respectively.

**Table 3:** Performance of natural language processing/machine learning models for fatigue domain by 3 symptom attributes.

Attributes and models		Precision (95% CI)	Sensitivity (95% CI)	Specificity (95% CI)	Accuracy (95% CI)	F1 (95% CI)	AUROCC^a^ (95% CI)	AUPRC^b^ (95% CI)
**Physical**								
	BERT^c^	0.593 (0.468-0.717)	0.427 (0.315-0.538)	0.929 (0.901-0.956)	0.832 (0.794-0.867)	0.496 (0.384-0.593)	0.775 (0.723-0.848)	0.537 (0.443-0.634)
	Word2vec/SVM^d^	0.600 (0.286-0.900)	0.073 (0.026-0.136)	0.988 (0.974-0.997)	0.810 (0.770-0.848)	0.130 (0.048-0.227)	0.726 (0.670-0.780)	0.375 (0.224-0.474)
	Word2vec/XGBoost^e^	0.595 (0.432-0.773)	0.268 (0.169-0.364)	0.956 (0.934-0.977)	0.822 (0.784-0.858)	0.370 (0.250-0.474)	0.726 (0.665-0.798)	0.461 (0.338-0.575)
**Cognitive**								
	BERT	0.803 (0.696-0.895)	0.757 (0.652-0.854)	0.963 (0.941-0.981)	0.929 (0.903-0.953)	0.779 (0.697-0.855)	0.948 (0.922-0.979)	0.855 (0.791-0.930)
	Word2vec/SVM	0.829 (0.690-0.946)	0.414 (0.292-0.535)	0.983 (0.968-0.994)	0.889 (0.861-0.917)	0.552 (0.418-0.657)	0.917 (0.886-0.951)	0.730 (0.632-0.855)
	Word2vec/XGBoost	0.767 (0.625-0.884)	0.471 (0.359-0.586)	0.972 (0.953-0.988)	0.889 (0.858-0.917)	0.584 (0.468-0.684)	0.860 (0.817-0.924)	0.659 (0.550-0.782)
**Social**								
	BERT	0.679 (0.500-0.848)	0.422 (0.289-0.568)	0.976 (0.960-0.990)	0.917 (0.891-0.943)	0.521 (0.379-0.658)	0.796 (0.704-0.912)	0.561 (0.434-0.741)
	Word2vec/SVM	0.778 (0.429-1.000)	0.156 (0.057-0.267)	0.995 (0.987-1.000)	0.905 (0.877-0.929)	0.259 (0.102-0.406)	0.817 (0.756-0.881)	0.393 (0.203-0.534)
	Word2vec/XGBoost	0.571 (0.286-0.833)	0.178 (0.068-0.300)	0.984 (0.971-0.995)	0.898 (0.868-0.924)	0.271 (0.118-0.415)	0.780 (0.706-0.850)	0.330 (0.154-0.436)

^a^AUROCC: area under the receiver operating characteristic curve.

^b^AUPRC: area under precision-recall curve.

^c^BERT: bidirectional encoder representations from transformers.

^d^SVM: support vector machine.

^e^XGBoost: extreme gradient boosting.

### Area Under the ROC Curves for Pain Interference and Fatigue

**Figure 2:**
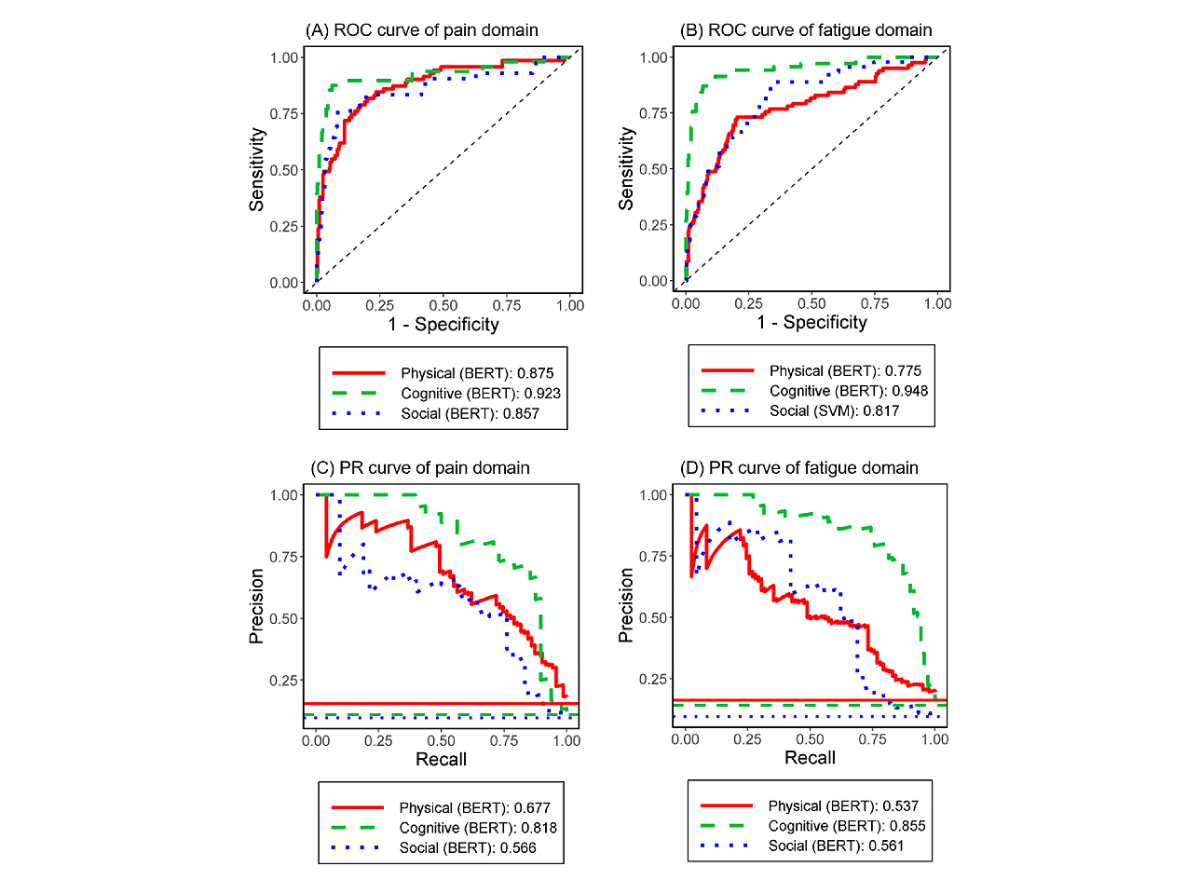
Area under the receiver operating characteristic curves and precision-recall curves for the best models of pain interference domain (left column) and fatigue domain (right column) by 3 symptom attributes. BERT: bidirectional encoder representations from transformers; PR: precision recall; ROC: receiver operating characteristic; SVM: support vector machine.

Figure 2 (upper) displays the specific NLP/ML method that had the highest area under the ROC curves for each attribute (detailed results in Tables 2 and 3). The diagonal line represents the random guess (ie, reference). For the pain interference domain (left panel), the BERT model was superior to the Word2vec/SVM and Word2vec/XGBoost models, and the areas under the ROC curve were 0.923 (95% CI 0.879-0.997) for cognitive, 0.875 (95% CI 0.824-0.948) for physical attributes, and 0.857 (95% CI 0.786-0.918) for social attributes. For the fatigue domain (right panel), the BERT model was superior to the Word2vec/SVM and Word2vec/XGBoost models, and areas under the ROC curve were (0.948, 95% CI 0.922-0.979) for cognitive and 0.775 (95% CI 0.723-0.848) for physical attributes. The values of BERT were significantly higher in identifying problems with cognitive attributes in both pain interference and fatigue domains compared with Word2vec/XGBoost (*P*<.05; Multimedia Appendix 13).

### Area Under the PR Curves for Pain Interference and Fatigue

Figure 2 (lower) displays the specific NLP/ML method that had the highest area under the PR curves for each attribute (see detailed results in Tables 2 and 3). The horizontal line at the bottom represents a random guess (ie, reference). For the pain interference domain (left panel), the BERT model was superior to the Word2vec/SVM and Word2vec/XGBoost models, and the areas under the PR curve were 0.818 (95% CI 0.735-0.917) for cognitive, 0.677 (95% CI 0.568-0.770) for physical attributes, and 0.566 (95% CI 0.402-0.750) for social attributes. For the fatigue domain (right panel), the BERT models were superior to the Word2vec/SVM and Word2vec/XGBoost models, and areas under the PR curve were 0.855 (95% CI 0.791-0.930) for cognitive, 0.561 (95% CI 0.434-0.741) for social attributes, and 0.537 (95% CI 0.443-0.634) for physical attributes. In addition, the values of BERT were significantly higher in identifying problems with cognitive and social attributes in both pain interference and fatigue domains compared with both Word2vec/SVM and Word2vec/XGBoost (*P*<.05; Multimedia Appendices 13 and 14).

## Discussion

### Principal Findings

Very limited studies have demonstrated the feasibility of applying NLP/ML methods to extract semantic features from unstructured PROs. This study applied different NLP/ML models to analyze PRO assessment in pediatric cancer survivorship, with a special focus on young survivors of pediatric cancer aged <18 years as a vulnerable population, and used rigorous methods to validate the performance of NLP/ML models. The results suggest that the BERT method outperformed the Word2vec/ML methods across different validation metrics in both the physical interference and fatigue symptom domains. Specifically, the BERT method yielded higher accuracy (>0.8), larger area under the ROC curve (>0.8, except for the social attribute in fatigue domain), and a larger area under the PR curve in identifying problems with all 3 attributes over 2 symptom domains compared with the Word2vec/SVM and Word2vec/XGBoost methods. The models with higher accuracy were characterized by high specificity (>0.9) but low sensitivity (<0.5) for all 3 attributes and 2 symptom domains.

The findings of high specificity and low sensitivity suggest that our NLP/ML algorithms can be used to identify problematic symptoms (ie, diagnostic confirmation) rather than for symptom screening. However, if the default threshold (ie, 0.5) for ROC curves was changed to a lower value that mimics the proportion of meaning units labeled as the presence of the problematic attribute, both specificity and sensitivity will reach the level of 0.7-0.8. How to use NLP/ML techniques to convert unstructured PROs into semantic features and transform the data into meaningful diagnostic information for clinical decision-making is an emerging topic [20,55]. It is important to extend our NLP/ML pipeline to assess other aspects of symptom problems (eg, severity and interference) for cancer populations and in a longitudinal context, which is valuable for detecting changes in symptom patterns and identifying early signs of adverse events [22,56,57].

### Comparisons of Model Performance

In both symptom domains, the performance of NLP/ML techniques (accuracy, F1 value, and areas under ROC and PR curves) in identifying problems with cognitive attributes was superior to physical and social attributes. Interestingly, model validity based on data collected from survivors and caregivers was slightly better than that of survivors alone (Multimedia Appendices 15 and 16). This finding is in part because of the inclusion of complementary information from survivors and caregivers and the increase in sample size.

The superior performance of NLP/ML techniques suggests the usefulness of interview-based methods for collecting unstructured PRO data to complement the survey-based methods that contain a prespecified fixed content of PROs in follow-up care among survivors of cancer. Using our validated NLP/ML algorithms to automatically abstract and label the semantic features of unstructured PROs derived from interviews represents an efficient strategy for collecting PRO data from busy clinics. Our NLP/ML approach can be extended to analyze other forms of unstructured PROs (eg, documented patient-clinician conversations and medical notes in EMRs) when data are available. Other novel technologies (eg, audio-recorded PROs) also deserve investigation in analyzing unstructured PROs. Multimodal sentiment analysis [58], which investigates affective states by extracting textual and audio features, can be combined with the semantic features from NLP to obtain a comprehensive understanding of survivors’ PROs. The successful application of NLP/ML for PRO assessment ideally requires the implementation of integrated platforms that interconnect the EHR-based medical note systems, NLP/ML analytics, and supportive tools for result display, clinical interpretation, and treatment recommendation [20,59-61]. The integrated platforms will facilitate clinicians in clinical decision-making for caring for survivors of cancer whose complex late medical effects can be predicted by the deterioration of symptoms and clinical parameters.

The superior performance of BERT to the Word2vec/ML method is because of the flexible design of BERT that accounts for contextual information of PROs. Basically, BERT includes multilayer deep neural networks (illustrated in self-attention layers of the fine-tuning process; Multimedia Appendix 5) to enable flexible feature extraction at different levels, such as syntactic, semantic, and contextual information. In comparison, Word2vec includes a one-level shallow neural network with limited flexibility. Uniquely, the semantic features derived by BERT capture different meanings of the same word in different contexts, whereas Word2vec generates static semantic features for each word that does not vary in different contexts.

### Different NLP Methods for Analyzing Unstructured PRO Data

The clinical application of NLP/ML in PRO research is still in its infancy. This study used the BERT model pretrained by Wikipedia and BooksCorpus to generate general semantic features as a starting point. The use of BooksCorpus and Wikipedia is appropriate for survivors of pediatric cancer, resulting in satisfactory model performance. This is because BooksCorpus and Wikipedia contain comprehensive generic terms that capture the heterogeneous health conditions experienced by various populations, including survivors of cancer, ranging from healthy (no late effects and no symptoms) to ill (severe late effects with severe symptoms). Alternatively, BERT models can be pretrained using larger free text data to generate comprehensive features of PROs. Similar methods may include SciBERT [62], trained by texts in Semantic Scholar; BioBERT [49], trained by texts in PubMed; and Clinical BERT [51], trained by clinical notes in MIMIC-III [63]. In addition, the health knowledge graph [64] can be used to integrate different concepts from various data elements in multiomics frameworks (including unstructured PROs in medical notes, structured PROs from patient survey, imaging, genetics, and treatment profiles), and analyze complex relationships among these data to improve evaluations of survivorship outcomes through a multitask learning framework [65].

### Limitations

This study contains several limitations. First, our samples were limited to survivors of pediatric cancer who were treated at a single institution. However, our samples represent diverse diagnoses, ages, races and ethnicities, and families residing in counties with poverty levels similar to the national average [15]. Second, we only analyzed pain interference and fatigue domains and restricted them to 3 key attributes of symptoms. Future studies are encouraged to apply our NLP/ML pipeline to analyze other PRO domains and include more comprehensive attribute classifications. Third, our data were collected cross-sectionally, which merely provides a snapshot of PROs. Future studies are needed to test the validity of abstracting longitudinal unstructured PROs to identify time-dependent patterns. In summary, we demonstrated a robust validity of NLP/ML algorithms in abstracting and analyzing unstructured PROs collected from interviews with childhood survivors of cancer and caregivers. These promising results suggest the utility of NLP/ML methods in future works for monitoring survivors’ PROs and the opportunity of extending our methods to other PRO domains and data collection systems (eg, audio-recorded or medical notes) under a unified platform that integrates EHR-based data collection systems, NLP/ML analytics, and supportive tools for interpretation of results and treatment recommendations. Integration of NLP/ML-based PRO assessment to complement other clinical data will facilitate the improvement of follow-up care for survivors of cancer.

## Appendix

Multimedia Appendix 1 Interview guides for pain interference domain (cancer survivor).

Multimedia Appendix 2 Interview guides for fatigue domain (cancer survivor).

Multimedia Appendix 3 Examples of meaning units derived from study participants and corresponding attributes.

Multimedia Appendix 4 The natural language processing/machine learning pipeline to analyze unstructured patient-reported outcomes data.

Multimedia Appendix 5 Concept of bidirectional encoder representations from transformers [base, uncased] techniques.

Multimedia Appendix 6 Tools or packages and fine-tuned hyper-parameters to analyze natural language processing/machine learning models.

Multimedia Appendix 7 Concept of Word2Vec techniques.

Multimedia Appendix 8 The changes of the area under the precision-recall curve among different natural language processing/machine learning models.

Multimedia Appendix 9 Performance of natural language processing/machine learning models for pain interference domain by three symptom attributes (cancer survivors and caregivers).

Multimedia Appendix 10 Performance of natural language processing/machine learning models for fatigue domain by three symptom attributes (cancer survivors and caregivers).

Multimedia Appendix 11 Five-fold cross-validation methods.

Multimedia Appendix 12 Frequency of attributes in pain interference and fatigue domains labeled by content experts.

Multimedia Appendix 13 The changes in the area under the receiver operating characteristic curve and precision-recall curve among different natural language processing/machine learning models (survivors and caregivers).

Multimedia Appendix 14 Precision-recall curves for pain interference and fatigue domains by three symptom attributes (survivors and caregivers).

Multimedia Appendix 15 Performance of natural language processing/machine learning models for pain interference domain by three symptom attributes (survivors only).

Multimedia Appendix 16 Performance of natural language processing/machine learning models for fatigue domain by three symptom attributes (survivors only).

## Abbreviations

BERT: bidirectional encoder representations from transformers
EMR: electronic medical record
ML: machine learning
NLP: natural language processing
PR: precision-recall
PRO: patient-reported outcome
PROMIS: Patient-Reported Outcomes Measurement Information System
ROC: receiver operating characteristic
SVM: support vector machine
TF-IDF: term frequency–inverse document frequency
XGBoost: extreme gradient boosting
