# Supplementary material for: Natural Language Processing and Machine Learning Methods to Characterize Unstructured Patient-Reported Outcomes: Validation Study
Source: J Med Internet Res. 2021 Nov 3;23(11):e26777. doi: 10.2196/26777 (PMC8600437; doi:10.2196/26777)
Supplement: Multimedia Appendix 1 [file jmir_v23i11e26777_app1.docx]

Table S1: Interview guides for pain interference domain (cancer survivor)

ID #________________________

| **PAIN INTERFERENCE**  **CHILD SEMI-STRUCTURED INTERVIEW (CANCER SURVIVOR)** | |
| --- | --- |
| **Introduction to the Interview**  **Hi, my name is _____________ and I’m a ________________ at ___________________.**  Thank you again for agreeing to do this interview with me today.  If it is ok, I will start the tape recorder.  [START TAPE RECORDER, VERBALLY RECORD:   - DATE & TIME OF INTERVIEW - FIRST NAME OF CHILD - YOUR NAME]   **VERBAL ASSENT**  CHILD’S NAME, we are glad that you want to do this interview and that your Mom (Dad/Parents) have given their permission for you to be in this study. It should about 30 minutes.  We are doing these interviews to learn more about what young people think and feel about their health.  Even though I will ask you some questions, this is not a test at all. We want to hear your ideas and experiences. There are no right or wrong answers, just what seems right to you.  We are tape recording and also taking notes. My notes help me keep track during the interview and the recording gives us a way to listen again if we need to. When this study is over we will destroy the tape recordings and our notes.  The questions I’m going to ask you are about how you think about health and things related to health, so some of them are personal or private. If you don’t want to answer a question, that’s fine. If you want to take a break or even stop, just let me know.  We will only use your first name during the interview and in our notes. I will not tell anyone here or in your family about anything that you said. Information from research is always private and confidential. There is only one exception. If you tell me that you are being harmed or might be harmed, then, together we will talk to someone to get the help you need to be safe.  Do you have any questions for me before we begin? | |
| **Rapport development**  Before we begin, I’d like to learn a little more about you…   - - *So how old are you now?*   - *What are some of your favorite things to do after school? And on the weekends?* | |
| **Statement of interview focus**  We want to talk with you about your experiences after stopping your cancer treatment. So, can you tell me when you were diagnosed with cancer? How old were you? [Write in the response here: ______________ years old.]  Now I want you to think about your health and life over the past two years.  First I would like to talk with you about how pain has affected you over the past two years. | |
| **Elicit reactions to key health words/phrases**  **Pain description:**   - First, let’s start by you telling me what you think about when you hear the word pain.   - How does pain feel to you?   - What are some words you use to describe your pain?   *Be sure that participant describes pain experiences, not just general experiences of feeling ill or uncomfortable.* | Notes |
| **General pain interference:**  Now I want you to think about all the things you do in your life:   - How did having pain affect what you could do in your life?   ***If no pain related to cancer experience, move on to another domain.* |  |
| **Pain experience #1: (Related to cancer survivorship)**  **Now let’s think about a specific time when you had a lot of pain. Remember we are asking you to think about the past two years. We are really interested in knowing how pain affects kids after they stop their cancer treatment.**   - - What things couldn’t you do that you normally would be able to do?   - How does pain affect the things you like to or the way that you have fun?   - What problems does the pain cause for you?   *If the participant does not talk about how pain has affected any parts of their life, proceed to ask the following more specific, open-ended questions.*  **Mobility interference:**   - How did the pain affect how you can move and get around?   **Emotional interference:**   - How does the pain affect the feelings that you have?   **Cognitive interference:**   - How does pain affect how you think?   **Social interference:**   - How does pain affect what you do with your friends and family?   **School interference:**   - How does pain affect what you do at school? |  |
| **Pain experience #2: (Related to Cancer survivorship)**  **Now let’s think about another time when you had a lot of pain after your cancer treatment.**  What are some things you couldn’t do in your life?   - - What things couldn’t you do that you normally would be able to do?   - How does pain affect the things you like to or the way that you have fun?   - What problems does the pain cause for you?   *If the participant does not talk about how pain has affected any parts of their life, proceed to ask the following more specific, open-ended questions.*  **Mobility interference:**   - How did the pain affect how you can move and get around?   **Emotional interference:**   - How does the pain affect the feelings that you have?   **Cognitive interference:**   - How does pain affect how you think?   **Social interference:**   - How does pain affect what you do with your friends and family?   **School interference:**   - How does pain affect what you do at school? |  |
