# Supplementary material for: Natural Language Processing and Machine Learning Methods to Characterize Unstructured Patient-Reported Outcomes: Validation Study
Source: J Med Internet Res. 2021 Nov 3;23(11):e26777. doi: 10.2196/26777 (PMC8600437; doi:10.2196/26777)
Supplement: Multimedia Appendix 2 [file jmir_v23i11e26777_app2.docx]

Table S2: Interview guides for fatigue domain (cancer survivor)

ID #________________________

| **FATIGUE**  **CHILD SEMI-STRUCTURED INTERVIEW (CANCER SURVIVOR)** | |
| --- | --- |
| **Introduction to the Interview**  **Hi, my name is _______________ and I’m a _______________ at ________________.**  Thank you again for agreeing to do this interview with me today.  If it is ok, I will start the tape recorder.  [START TAPE RECORDER, VERBALLY RECORD:   - DATE & TIME OF INTERVIEW - FIRST NAME OF CHILD - YOUR NAME]   **VERBAL ASSENT**  CHILD’S NAME, we are glad that you want to do this interview and that your Mom (Dad/Parents) have given their permission for you to be in this study. It should take about 30 minutes.  We are doing these interviews to learn more about what young people think and feel about their health.  Even though I will ask you some questions, this is not a test at all. We want to hear your ideas and experiences. There are no right or wrong answers, just what seems right to you.  We are tape recording and also taking notes. My notes help me keep track during the interview and the recording gives us a way to listen again if we need to. When this study is over we will destroy the tape recordings and our notes.  The questions I’m going to ask you are about what you think about health and things related to health, so some of them are personal or private. If you don’t want to answer a question, that’s fine. If you want to take a break or even stop, just let me know.  We will only use your first name during the interview and in our notes. I will not tell anyone here or in your family about anything that you said. Information from research is always private and confidential. There is only one exception. If you tell me that you are being harmed or might be harmed, then, together we will talk to someone to get the help you need to be safe.  Do you have any questions for me before we begin? | |
| **Rapport development**  Before we begin, I’d like to learn a little more about you…   - - *So how old are you now?*   - *What are some of your favorite things to do after school? And on the weekends?* | |
| **Statement of interview focus**  We want to talk with you about your experiences after stopping your cancer treatment. So, can you tell me when you were diagnosed with cancer? How old were you? [Write in the response here: ______________ years old.]  Now I want you to think about your life over the past two years.  First I would like to talk with you about how being tired has affected you over the past two years. | |
| **Elicit reactions to key health words/phrases**  **Fatigue description:**   - First, let’s start by you telling me what you think about when you hear the word ‘fatigue’ or ‘being tired’.   - How does being tired feel to you?   - What are some words you use to describe being tired? | Notes: |
| **General fatigue interference:**  Now I want you to think about all the things you do in your life:   - How does being tired affect what you can do in your life? |  |
| **Fatigue experience #1: (Related to surviving cancer)**  **Now let’s think about a specific time when you extremely tired. Remember we are asking you to think about the past two years. We are really interested in knowing how being tired affects kids after they stop their cancer treatment.**   - - What things couldn’t you do that you normally would be able to do?   - How does being tired affect the things you like to or the way that you have fun?   - What problems do you have from being very tired?   *If the participant does not talk about how fatigue has affected any parts of their life, proceed to ask the following more specific, open-ended questions. If no experiences of fatigue, move on to another domain.*  **Mobility interference:**   - How did being tired affect how you can move and get around?   **Emotional interference:**   - How does being tired affect the feelings that you have?   **Cognitive interference:**   - How does being tired affect how you think?   **Social interference:**   - How does being tired affect what you do with your friends and family?   **School interference:**   - How does being tired affect what you do at school? |  |
| **Fatigue experience #2: (Related to surviving cancer)**  **Now let’s think about another time when you were extremely tired in the time since your cancer treatment. What are some things you couldn’t do in your life?**   - - What things couldn’t you do that you normally would be able to do?   - How does being tired affect the things you like to or the way that you have fun?   - What problems do you have from being very tired?   *If the participant does not talk about how fatigue has affected any parts of their life, proceed to ask the following more specific, open-ended questions.*  **Mobility interference:**   - How did being tired affect how you can move and get around?   **Emotional interference:**   - How does being tired affect the feelings that you have?   **Cognitive interference:**   - How does being tired affect how you think?   **Social interference:**   - How does being tired affect what you do with your friends and family?   **School interference:**   - How does being tired affect what you do at school? |  |
