# Supplementary material for: Natural Language Processing and Machine Learning Methods to Characterize Unstructured Patient-Reported Outcomes: Validation Study
Source: J Med Internet Res. 2021 Nov 3;23(11):e26777. doi: 10.2196/26777 (PMC8600437; doi:10.2196/26777)
Supplement: Multimedia Appendix 3 [file jmir_v23i11e26777_app3.docx]

Table S3**:** Examples of meaning units derived from study participants and corresponding attributes

| Pain interference domain |  |  |
| --- | --- | --- |
| Meaning unit | Meaningful concepts | Attributes |
| Can’t play, and go outside when I have a headache. | Hard to do sports or exercise when had pain | Physical |
| Like when I stand up, it feels like it’s the pain in my head going down and it starts to hurt more. | Hard to stay  standing when had pain | Physical |
| I can't like learn. Like I can't pay attention and stuff at school, when I have a headache. | Hard to pay attention  when had pain | Cognitive |
| Those are two slots that I cannot fill with other thoughts. | hard to think when had pain | Cognitive |
| I actually had to leave school a few times because it ovary pain] was so bad. | Missed school  when had pain | Social |
| Well it affected me because most of the time I had to stay indoors and I couldn’t really go out with friends and yeah. | Hard to have fun with  friends because had pain | Social |
| Fatigue Domain |  |  |
| Meaning unit | Meaningful concepts | Attributes |
| Like when I… push up my arms during gymnastics start to wobble. | Too tired to do sports or exercise | Physical |
| Normally I feel like when I get home I can go outside and usually I can ride my four-wheeler afterschool and during the school year... I can’t because I’m just so tired. | Too tired to do things outside | Physical |
| It's hard to get my school work done when I'm tired. | Hard to keep up with schoolwork | Cognitive |
| I can’t pay attention. And it’s hard for me to stay awake in school. | Too tired and hard to pay attention | Cognitive |
| I guess I'm not as fun and exciting to friends and family whenever I'm tired. | Kept me from having fun | Social |
| I wanted to lay on the couch and be able to sleep instead of going out with family and friends after playing softball games. | Too tired to spend time with friends | Social |
