# Supplementary material for: Natural Language Processing and Machine Learning Methods to Characterize Unstructured Patient-Reported Outcomes: Validation Study
Source: J Med Internet Res. 2021 Nov 3;23(11):e26777. doi: 10.2196/26777 (PMC8600437; doi:10.2196/26777)
Supplement: Multimedia Appendix 4 [file jmir_v23i11e26777_app4.docx]

Figure S1: The NLP/ML pipeline to analyze unstructured PROs data


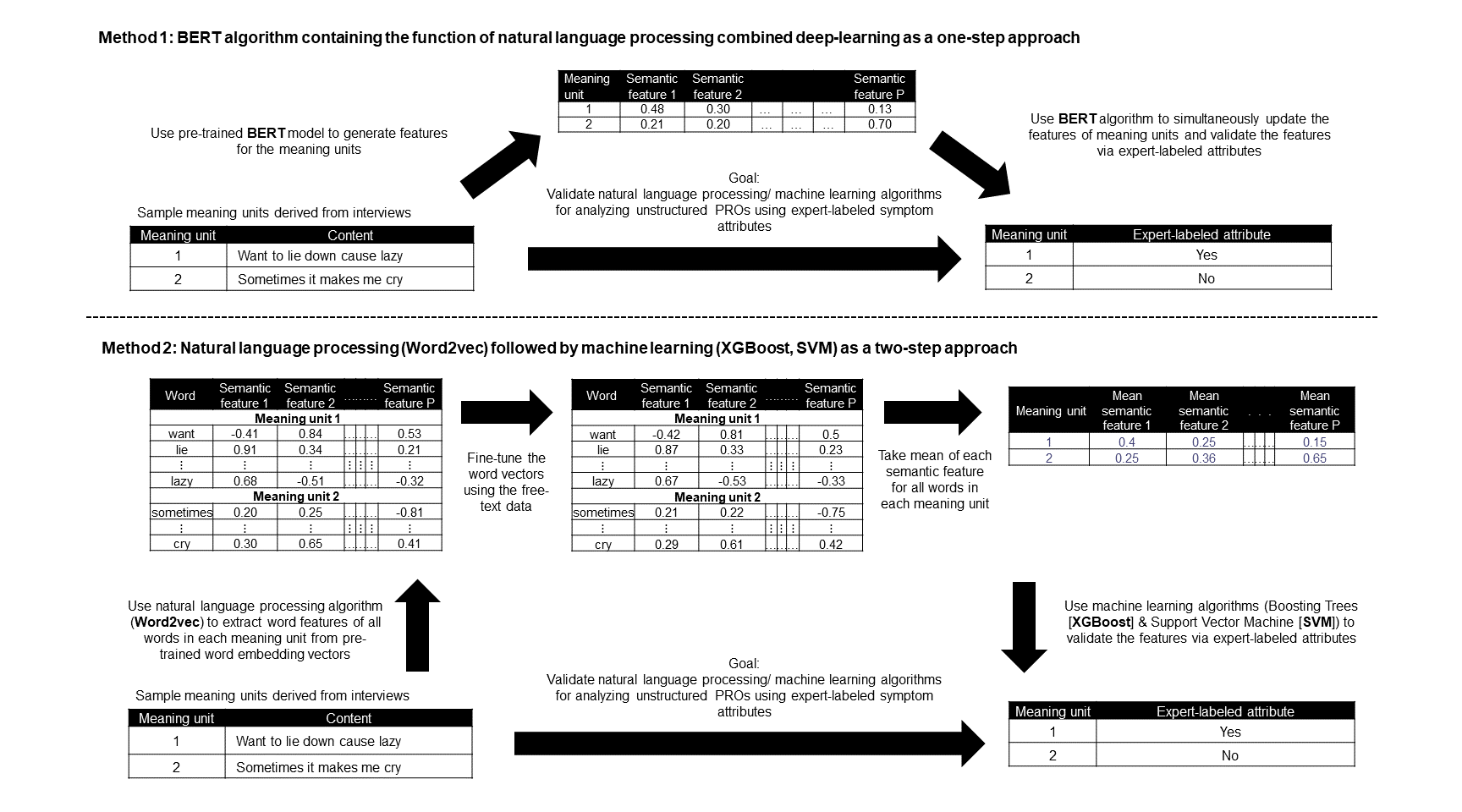


Abbreviations:

BERT, Bidirectional Encoder Representations from Transformers; NLP, natural language processing; ML, machine learning; PROs, patient-reported outcomes; SVM, Support Vector Machine; XGBoost, eXtreme Gradient Boosting
