# Supplementary material for: Natural Language Processing and Machine Learning Methods to Characterize Unstructured Patient-Reported Outcomes: Validation Study
Source: J Med Internet Res. 2021 Nov 3;23(11):e26777. doi: 10.2196/26777 (PMC8600437; doi:10.2196/26777)
Supplement: Multimedia Appendix 5 [file jmir_v23i11e26777_app5.docx]

Figure S2: Concept of BERT [base, uncased] techniques


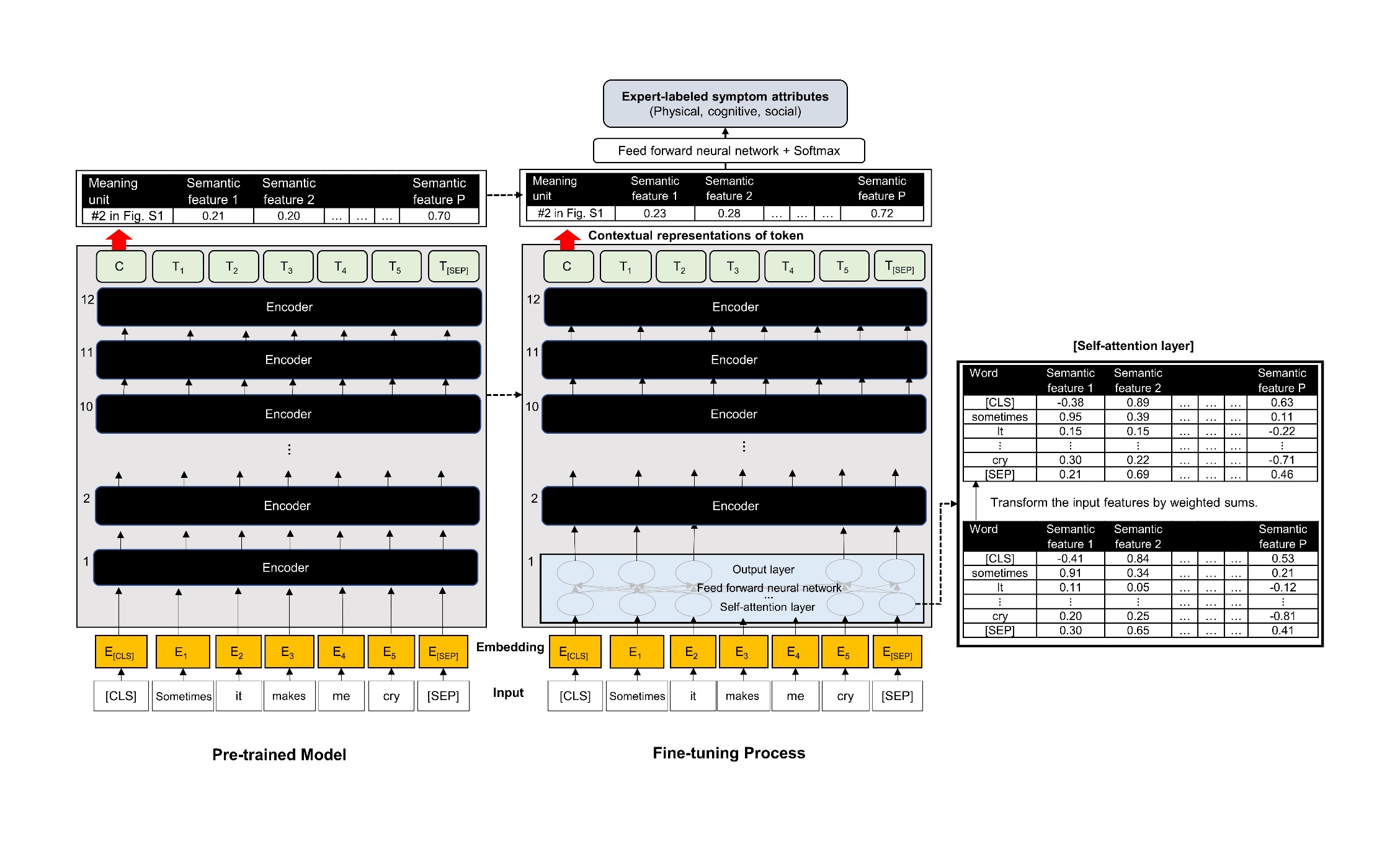


Footnote:

The pre-trained model is the BERT [base, uncased] model obtained from the huggingface model repository (<https://huggingface.co/bert-base-uncased>), which was an pytorch implementation of the base, uncased BERT model in Devlin J, Chang M-W, Lee K, et al (2018).

The BERT [base, uncased] model was pre-trained by the BooksCorpus (800M words) used in Zhu, Kiros, Zemel, et al (2015), and English Wikipedia (2,500M words) used in Devlin J, Chang M-W, Lee K, et al (2018).

Feed forward neural network: the node in the previous layer is fully connected to the node in the next layer.

Encoder: a deep neural network consists of a self-attention layer and a feed forward neural network layer.

[CLS] is a special symbol added in front of every input example, and [SEP] is a special separator token (e.g. connected two sentences).

The features of word embeddings are fed to the BERT encoder as input.

The weights used for calculating the weighted sums are based on the context, which enable context-dependent word features.
