# Supplementary material for: Natural Language Processing and Machine Learning Methods to Characterize Unstructured Patient-Reported Outcomes: Validation Study
Source: J Med Internet Res. 2021 Nov 3;23(11):e26777. doi: 10.2196/26777 (PMC8600437; doi:10.2196/26777)
Supplement: Multimedia Appendix 6 [file jmir_v23i11e26777_app6.docx]

Table S4: Tools/packages and fine-tuned hyper-parameters to analyze NLP/ML models

| NLP/ML models | Tools/packages | Fine-tuned hyper-parameters |
| --- | --- | --- |
| TF-IDF | TfidfVectorizer in python module sklearn | Some of the features are highly correlated. Hence, singular value decomposition is used to generate a new set of features. The number of features is determined so that the variance of the new features explains 90% of that of the original features. |
| GloVe | GloVe, Mittens in python module mittens  Pre-trained model from glove.6B.300d.txt in  https://nlp.stanford.edu/projects/glove/ | In Mittens   - n=300 - max_iter=1000 |
| Word2vec/SVM | Caret R package  svmLinear method | - Tuned by a grid search with exp(-4), exp(-3.5), exp(-3),… exp(6). - Features pre-processed with centering and scaling. |
| Word2vec/XGBoost | Caret R package xgbTree method | A random search to identify a possible region and then fine-tune the parameters with grid search   - Shrinkage (eta); - Number of Boosting Iterations (nrounds); - Max Tree Depth (max_depth); - Subsample Ratio of Columns (colsample_bytree); - Minimum Sum of Instance Weight (min_child_weight). |
| BERT | BertForSequenceClassification  in pytorch_transformers  AdamW optimizer | - max_seq_length = 128 - train_batch_size = 20 - eval_batch_size = 20 - learning_rate = 4e-5 - adam_epsilon = 1e-8 - max_grad_norm = 1.0 - num_train_epochs = 10 |

Abbreviations:

BERT, Bidirectional Encoder Representations from Transformers; GloVe, Global Vectors for Word Representation; ML, Machine learning; NLP, Natural Language Processing; SVM, Support Vector Machine; TF-IDF, Term Frequency–Inverse Document Frequency; XGBoost, eXtreme Gradient Boosting
