# Supplementary material for: Natural Language Processing and Machine Learning Methods to Characterize Unstructured Patient-Reported Outcomes: Validation Study
Source: J Med Internet Res. 2021 Nov 3;23(11):e26777. doi: 10.2196/26777 (PMC8600437; doi:10.2196/26777)
Supplement: Multimedia Appendix 7 [file jmir_v23i11e26777_app7.docx]

Figure S3: Concept of Word2Vec techniques


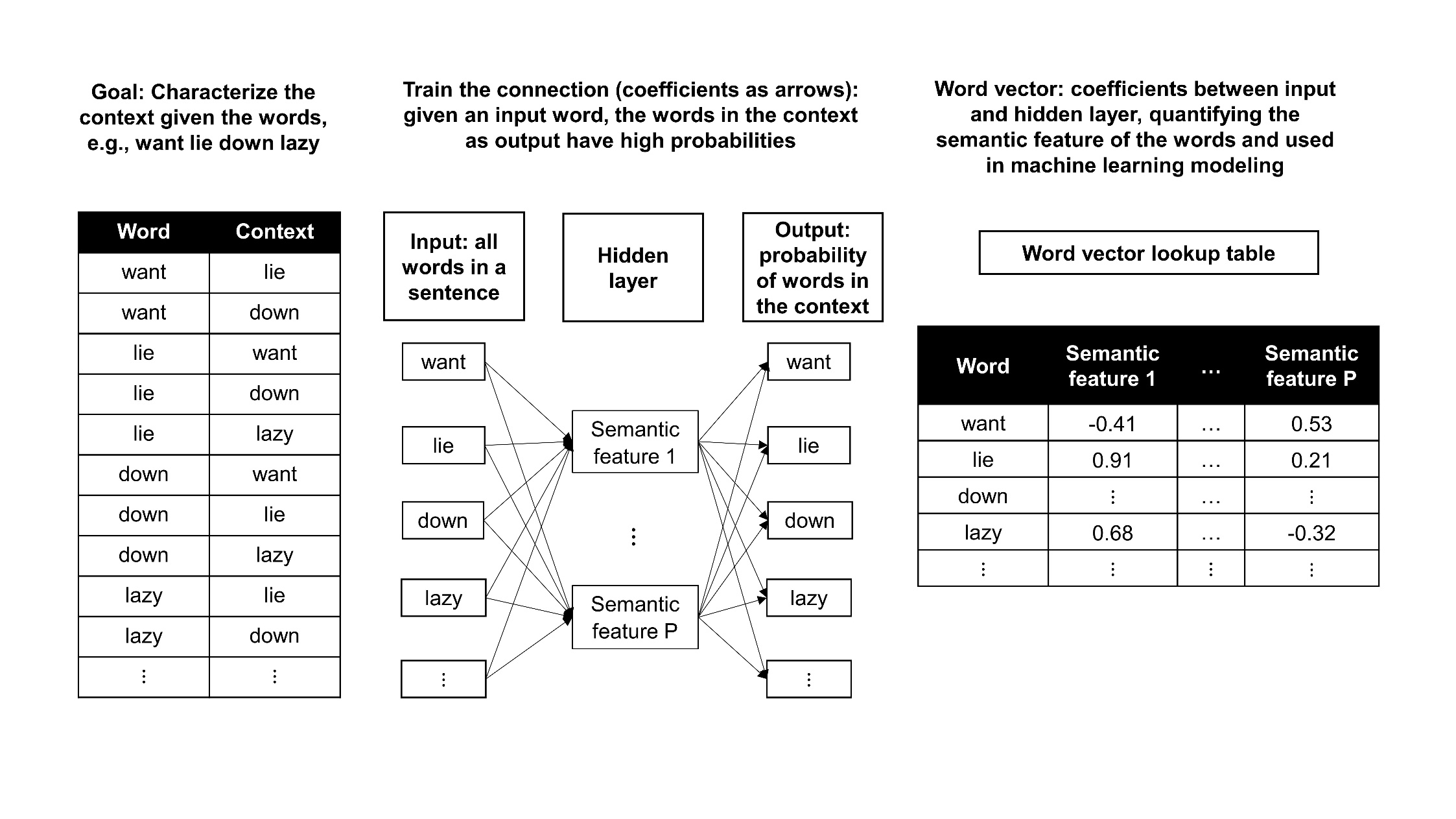


Footnote:

Word, input of the predictive model; Context, the words around the input word (also known as context window), which are the output of the predictive model and predicted by the input word; Semantic feature/ hidden layer, the distinct underlying characteristics that capture the semantic similarities of words appear in the same context, i.e., near each other.
