# Supplementary material for: Natural Language Processing and Machine Learning Methods to Characterize Unstructured Patient-Reported Outcomes: Validation Study
Source: J Med Internet Res. 2021 Nov 3;23(11):e26777. doi: 10.2196/26777 (PMC8600437; doi:10.2196/26777)
Supplement: Multimedia Appendix 8 [file jmir_v23i11e26777_app8.docx]

Table S5: The changes of the area under the precision-recall curve among different NLP/ML models

| Domains | Attributes | GloVe/SVM vs. Word2vec/SVM | GloVe/XGBoost vs. Word2vec/XGBoost | BERT vs.  TF-IDF | BERT vs.  GloVe/SVM | BERT vs. GloVe/XGBoost | BERT vs. BioBERT | BERT vs. BlueBERT | BERT vs. Clinical BERT |
| --- | --- | --- | --- | --- | --- | --- | --- | --- | --- |
|  |  | Changes in AUPRC  (95% CI) | Changes in AUPRC  (95% CI) | Changes in AUPRC  (95% CI) | Changes in AUPRC  (95% CI) | Changes in AUPRC  (95% CI) | Changes in AUPRC  (95% CI) | Changes in AUPRC  (95% CI) | Changes in AUPRC  (95% CI) |
| Pain inter-ference | Physical | 0.117  (0.050, 0.190) | 0.045  (-0.043, 0.149) | 0.250  (0.116, 0.381) | 0.171  (0.043, 0.285) | 0.169  (0.050, 0.279) | 0.159  (0.042, 0.271) | 0.085  (-0.017, 0.183) | 0.142  (0.028, 0.244) |
|  | Cognitive | -0.074  (-0.181, 0.033) | -0.186  (-0.322, -0.042) | 0.448  (0.305, 0.573) | 0.135  (0.004, 0.255) | 0.158  (0.043, 0.288) | 0.165  (0.043, 0.274) | 0.084  (0.004, 0.168) | 0.217  (0.094, 0.334) |
|  | Social | -0.043  (-0.135, 0.035) | -0.105  (-0.241, 0.065) | 0.375  (0.230, 0.515) | 0.204  (0.059, 0.344) | 0.147  (0.023, 0.290) | 0.020  (-0.127, 0.157) | -0.008  (-0.133, 0.115) | 0.092  (-0.051, 0.237) |
| Fatigue | Physical | -0.034  (-0.118, 0.031) | 0.051  (-0.029, 0.133) | 0.165  (0.014, 0.266) | 0.093  (0.001, 0.202) | 0.094  (0.012, 0.195) | 0.018  (-0.091, 0.089) | 0.040  (-0.094, 0.113) | 0.019  (-0.101, 0.093) |
|  | Cognitive | 0.039  (-0.079, 0.139) | -0.027  (-0.125, 0.077) | 0.302  (0.169, 0.409) | 0.164  (0.035, 0.280) | 0.169  (0.075, 0.263) | 0.033  (-0.048, 0.121) | 0.066  (-0.022, 0.151) | 0.015  (-0.054, 0.088) |
|  | Social | -0.033  (-0.101, 0.066) | 0.063  (-0.048, 0.159) | 0.285  (0.148, 0.430) | 0.135  (0.019, 0.272) | 0.294  (0.137, 0.432) | 0.179  (0.047, 0.329) | 0.061  (-0.070, 0.193) | 0.109  (-0.032, 0.264) |

Abbreviations:

AUPRC, area under precision-recall curve; BERT, Bidirectional Encoder Representations from Transformers; BioBERT, BERT for Biomedical Text Mining; BlueBERT, Biomedical Language Understanding Evaluation BERT; CI, confidence interval; GloVe, Global Vectors for Word Representation; ML, machine learning; NLP, natural language processing; SVM, Support Vector Machine; TF-IDF, Term Frequency–Inverse Document Frequency; XGBoost, eXtreme Gradient Boosting
