# Supplementary material for: Natural Language Processing and Machine Learning Methods to Characterize Unstructured Patient-Reported Outcomes: Validation Study
Source: J Med Internet Res. 2021 Nov 3;23(11):e26777. doi: 10.2196/26777 (PMC8600437; doi:10.2196/26777)
Supplement: Multimedia Appendix 9 [file jmir_v23i11e26777_app9.docx]

Table S6: Performance of NLP/ML models for pain interference domain by three symptom attributes (cancer survivors and caregivers)

| Attributes | Models | Precision  (95% CI) | Sensitivity  (95% CI) | Specificity  (95% CI) | Accuracy  (95% CI) | F1  (95% CI) | AUROCC  (95% CI) | AUPRC  (95% CI) |
| --- | --- | --- | --- | --- | --- | --- | --- | --- |
| Physical | TF-IDF/  SVM | 0.727  (0.444, 1.000) | 0.113  (0.044, 0.195) | 0.991  (0.979, 1.000) | 0.831  (0.793, 0.867) | 0.195  (0.080, 0.314) | 0.719  (0.630, 0.798) | 0.427  (0.263, 0.543) |
|  | Glove/SVM | 0.609  (0.400, 0.808) | 0.197  (0.108, 0.284) | 0.972  (0.953, 0.988) | 0.831  (0.795, 0.867) | 0.298  (0.175, 0.404) | 0.792  (0.745, 0.843) | 0.506  (0.377, 0.635) |
|  | Glove/  XGboost | 0.773  (0.583, 0.944) | 0.239  (0.141, 0.344) | 0.984  (0.969, 0.997) | 0.849  (0.811, 0.882) | 0.366  (0.233, 0.486) | 0.769  (0.706, 0.851) | 0.508  (0.375, 0.624) |
|  | BioBERT | 0.623  (0.479, 0.754) | 0.465  (0.359, 0.587) | 0.938  (0.909, 0.963) | 0.852  (0.813, 0.887) | 0.532  (0.424, 0.638) | 0.752  (0.681, 0.838) | 0.518  (0.377, 0.646) |
|  | BlueBERT | 0.750  (0.622, 0.865) | 0.507  (0.384, 0.627) | 0.963  (0.941, 0.981) | 0.880  (0.849, 0.913) | 0.605  (0.492, 0.708) | 0.820  (0.746, 0.876) | 0.592  (0.437, 0.698) |
|  | Clinical BERT | 0.634  (0.476, 0.775) | 0.366  (0.254, 0.475) | 0.953  (0.928, 0.975) | 0.847  (0.811, 0.880) | 0.464  (0.349, 0.569) | 0.787  (0.708, 0.841) | 0.535  (0.367, 0.678) |
| Cognitive | TF-IDF/  SVM | 0.588  (0.333, 0.818) | 0.208  (0.106, 0.333) | 0.98  (0.965, 0.994) | 0.885  (0.854, 0.913) | 0.308  (0.169, 0.452) | 0.698  (0.598, 0.782) | 0.370  (0.241, 0.507) |
|  | Glove/SVM | 0.833  (0.667, 0.962) | 0.417  (0.273, 0.562) | 0.988  (0.977, 0.997) | 0.918  (0.890, 0.944) | 0.556  (0.395, 0.692) | 0.910  (0.871, 0.954) | 0.683  (0.568, 0.832) |
|  | Glove/  XGboost | 0.688  (0.454, 0.900) | 0.229  (0.115, 0.354) | 0.985  (0.971, 0.997) | 0.893  (0.862, 0.921) | 0.344  (0.192, 0.486) | 0.893  (0.839, 0.943) | 0.660  (0.558, 0.788) |
|  | BioBERT | 0.758  (0.600, 0.895) | 0.521  (0.395, 0.679) | 0.977  (0.959, 0.991) | 0.921  (0.895, 0.946) | 0.617  (0.487, 0.742) | 0.891  (0.838, 0.961) | 0.653  (0.529, 0.793) |
|  | BlueBERT | 1  (1, 1) | 0.479  (0.349, 0.625) | 1  (1, 1) | 0.936  (0.910, 0.959) | 0.648  (0.517, 0.769) | 0.862  (0.802, 0.927) | 0.734  (0.622, 0.829) |
|  | Clinical BERT | 0.786  (0.611, 0.926) | 0.458  (0.322, 0.606) | 0.983  (0.966, 0.994) | 0.918  (0.887, 0.944) | 0.579  (0.441, 0.711) | 0.833  (0.767, 0.935) | 0.601  (0.472, 0.738) |
| Social | TF-IDF/  SVM | 0  (0, 0) | 0  (0, 0) | 0.997  (0.991, 1.000) | 0.89  (0.859, 0.921) | NA  (NA, NA) | 0.640  (0.539, 0.763) | 0.191  (0.085, 0.267) |
|  | Glove/SVM | 0.333  (0, 1.000) | 0.024  (0, 0.081) | 0.994  (0.985, 1.000) | 0.890  (0.857, 0.923) | 0.044  (0.036, 0.158) | 0.818  (0.764, 0.892) | 0.352  (0.152, 0.464) |
|  | Glove/  XGboost | 0.714  (0.333, 1.000) | 0.119  (0.03, 0.220) | 0.994  (0.986, 1.000) | 0.900  (0.870, 0.931) | 0.204  (0.059, 0.348) | 0.811  (0.745, 0.883) | 0.409  (0.248, 0.544) |
|  | BioBERT | 0.643  (0.454, 0.818) | 0.429  (0.286, 0.583) | 0.971  (0.951, 0.988) | 0.913  (0.882, 0.941) | 0.514  (0.371, 0.651) | 0.839  (0.780, 0.927) | 0.546  (0.387, 0.706) |
|  | BlueBERT | 0.741  (0.556, 0.893) | 0.476  (0.326, 0.622) | 0.980  (0.963, 0.992) | 0.926  (0.898, 0.949) | 0.580  (0.426, 0.704) | 0.835  (0.751, 0.904) | 0.574  (0.406, 0.76) |
|  | Clinical BERT | 0.538  (0.333, 0.739) | 0.333  (0.194, 0.476) | 0.966  (0.944, 0.983) | 0.898  (0.864, 0.928) | 0.412  (0.254, 0.556) | 0.812  (0.732, 0.931) | 0.474  (0.281, 0.706) |

Abbreviations:

AUPRC, area under precision-recall curve; AUROCC, area under the receiver operating characteristic curve; BERT, Bidirectional Encoder Representations from Transformers; BioBERT, BERT for Biomedical Text Mining; BlueBERT, Biomedical Language Understanding Evaluation BERT; CI, confidence interval; GloVe, Global Vectors for Word Representation; ML, machine learning; NLP, natural language processing; SVM, Support Vector Machine; TF-IDF, Term Frequency–Inverse Document Frequency; XGBoost, eXtreme Gradient Boosting
