# Supplementary material for: Natural Language Processing and Machine Learning Methods to Characterize Unstructured Patient-Reported Outcomes: Validation Study
Source: J Med Internet Res. 2021 Nov 3;23(11):e26777. doi: 10.2196/26777 (PMC8600437; doi:10.2196/26777)
Supplement: Multimedia Appendix 10 [file jmir_v23i11e26777_app10.docx]

Table S7: Performance of NLP/ML models for fatigue domain by three symptom attributes (cancer survivors and caregivers)

| Attributes | Models | Precision  (95% CI) | Sensitivity  (95% CI) | Specificity  (95% CI) | Accuracy  (95% CI) | F1  (95% CI) | AUROCC  (95% CI) | AUPRC  (95% CI) |
| --- | --- | --- | --- | --- | --- | --- | --- | --- |
| Physical | TF-IDF/  SVM | 0.409  (0.200, 0.625) | 0.110  (0.047, 0.176) | 0.962  (0.940, 0.980) | 0.797  (0.757, 0.832) | 0.173  (0.078, 0.268) | 0.702  (0.646, 0.761) | 0.372  (0.284, 0.477) |
|  | Glove/SVM | 0.667  (0.250, 1.000) | 0.049  (0.011, 0.099) | 0.994  (0.985, 1.000) | 0.811  (0.773, 0.849) | 0.091  (0.023, 0.176) | 0.750  (0.696, 0.805) | 0.409  (0.292, 0.510) |
|  | Glove/  XGboost | 0.543  (0.371, 0.704) | 0.232  (0.148, 0.329) | 0.953  (0.931, 0.974) | 0.813  (0.775, 0.851) | 0.325  (0.218, 0.434) | 0.713  (0.645, 0.772) | 0.408  (0.262, 0.513) |
|  | BioBERT | 0.560  (0.451, 0.667) | 0.512  (0.407, 0.614) | 0.903  (0.871, 0.933) | 0.827  (0.787, 0.863) | 0.535  (0.435, 0.626) | 0.783  (0.737, 0.85) | 0.519  (0.374, 0.619) |
|  | BlueBERT | 0.506  (0.400, 0.622) | 0.488  (0.390, 0.589) | 0.886  (0.853, 0.918) | 0.809  (0.771, 0.844) | 0.497  (0.403, 0.583) | 0.792  (0.738, 0.855) | 0.497  (0.384, 0.617) |
|  | Clinical BERT | 0.582  (0.459, 0.706) | 0.476  (0.370, 0.58) | 0.918  (0.888, 0.947) | 0.832  (0.797, 0.868) | 0.523  (0.420, 0.616) | 0.790  (0.715, 0.840) | 0.518  (0.417, 0.638) |
| Cognitive | TF-IDF/  SVM | 0.742  (0.568, 0.889) | 0.329  (0.221, 0.453) | 0.977  (0.960, 0.992) | 0.870  (0.837, 0.903) | 0.455  (0.330, 0.579) | 0.824  (0.767, 0.871) | 0.553  (0.434, 0.691) |
|  | Glove/SVM | 0.935  (0.840, 1.000) | 0.414  (0.299, 0.542) | 0.994  (0.985, 1.000) | 0.898  (0.870, 0.927) | 0.574  (0.447, 0.692) | 0.913  (0.882, 0.953) | 0.691  (0.565, 0.807) |
|  | Glove/  XGboost | 0.703  (0.548, 0.842) | 0.371  (0.261, 0.487) | 0.969  (0.949, 0.986) | 0.870  (0.835, 0.901) | 0.486  (0.360, 0.594) | 0.899  (0.864, 0.941) | 0.686  (0.571, 0.791) |
|  | BioBERT | 0.860  (0.768, 0.943) | 0.700  (0.584, 0.803) | 0.977  (0.962, 0.992) | 0.931  (0.908, 0.955) | 0.772  (0.686, 0.844) | 0.938  (0.901, 0.989) | 0.822  (0.731, 0.936) |
|  | BlueBERT | 0.872  (0.762, 0.962) | 0.586  (0.464, 0.696) | 0.983  (0.968, 0.994) | 0.917  (0.889, 0.941) | 0.701  (0.593, 0.788) | 0.879  (0.825, 0.947) | 0.789  (0.706, 0.902) |
|  | Clinical BERT | 0.793  (0.680, 0.902) | 0.657  (0.543, 0.767) | 0.966  (0.946, 0.985) | 0.915  (0.887, 0.941) | 0.719  (0.621, 0.800) | 0.949  (0.924, 0.990) | 0.840  (0.773, 0.904) |
| Social | TF-IDF/  SVM | 0.636  (0.333, 0.909) | 0.156  (0.061, 0.268) | 0.989  (0.978, 0.997) | 0.901  (0.872, 0.927) | 0.250  (0.107, 0.393) | 0.636  (0.558, 0.702) | 0.276  (0.130, 0.393) |
|  | Glove/SVM | 0.600  (0.250, 0.900) | 0.133  (0.044, 0.244) | 0.989  (0.978, 0.997) | 0.898  (0.870, 0.924) | 0.218  (0.081, 0.369) | 0.833  (0.783, 0.898) | 0.426  (0.303, 0.582) |
|  | Glove/  XGboost | 0.400  (0.167, 0.684) | 0.133  (0.044, 0.245) | 0.976  (0.960, 0.990) | 0.887  (0.856, 0.917) | 0.200  (0.073, 0.348) | 0.757  (0.679, 0.841) | 0.267  (0.148, 0.361) |
|  | BioBERT | 0.650  (0.444, 0.850) | 0.289  (0.167, 0.419) | 0.981  (0.966, 0.995) | 0.908  (0.879, 0.934) | 0.400  (0.250, 0.533) | 0.743  (0.669, 0.878) | 0.382  (0.255, 0.499) |
|  | BlueBERT | 0.600  (0.375, 0.810) | 0.267  (0.133, 0.400) | 0.979  (0.965, 0.992) | 0.903  (0.875, 0.929) | 0.369  (0.200, 0.51) | 0.788  (0.714, 0.891) | 0.5  (0.351, 0.665) |
|  | Clinical BERT | 0.652  (0.450, 0.850) | 0.333  (0.200, 0.476) | 0.979  (0.963, 0.992) | 0.910  (0.882, 0.936) | 0.441  (0.286, 0.576) | 0.738  (0.643, 0.828) | 0.452  (0.296, 0.630) |

Abbreviations:

AUPRC, area under precision-recall curve; AUROCC, area under the receiver operating characteristic curve; BERT, Bidirectional Encoder Representations from Transformers; BioBERT, BERT for Biomedical Text Mining; BlueBETR, Biomedical Language Understanding Evaluation BERT; CI, confidence interval; GloVe, Global Vectors for Word Representation; ML, machine learning; NLP, natural language processing; SVM, Support Vector Machine; TF-IDF, Term Frequency–Inverse Document Frequency; XGBoost, eXtreme Gradient Boosting
