# Supplementary material for: Natural Language Processing and Machine Learning Methods to Characterize Unstructured Patient-Reported Outcomes: Validation Study
Source: J Med Internet Res. 2021 Nov 3;23(11):e26777. doi: 10.2196/26777 (PMC8600437; doi:10.2196/26777)
Supplement: Multimedia Appendix 11 [file jmir_v23i11e26777_app11.docx]

Figure S4: 5-fold cross-validation methods

1. 5-fold cross-validation for BERT


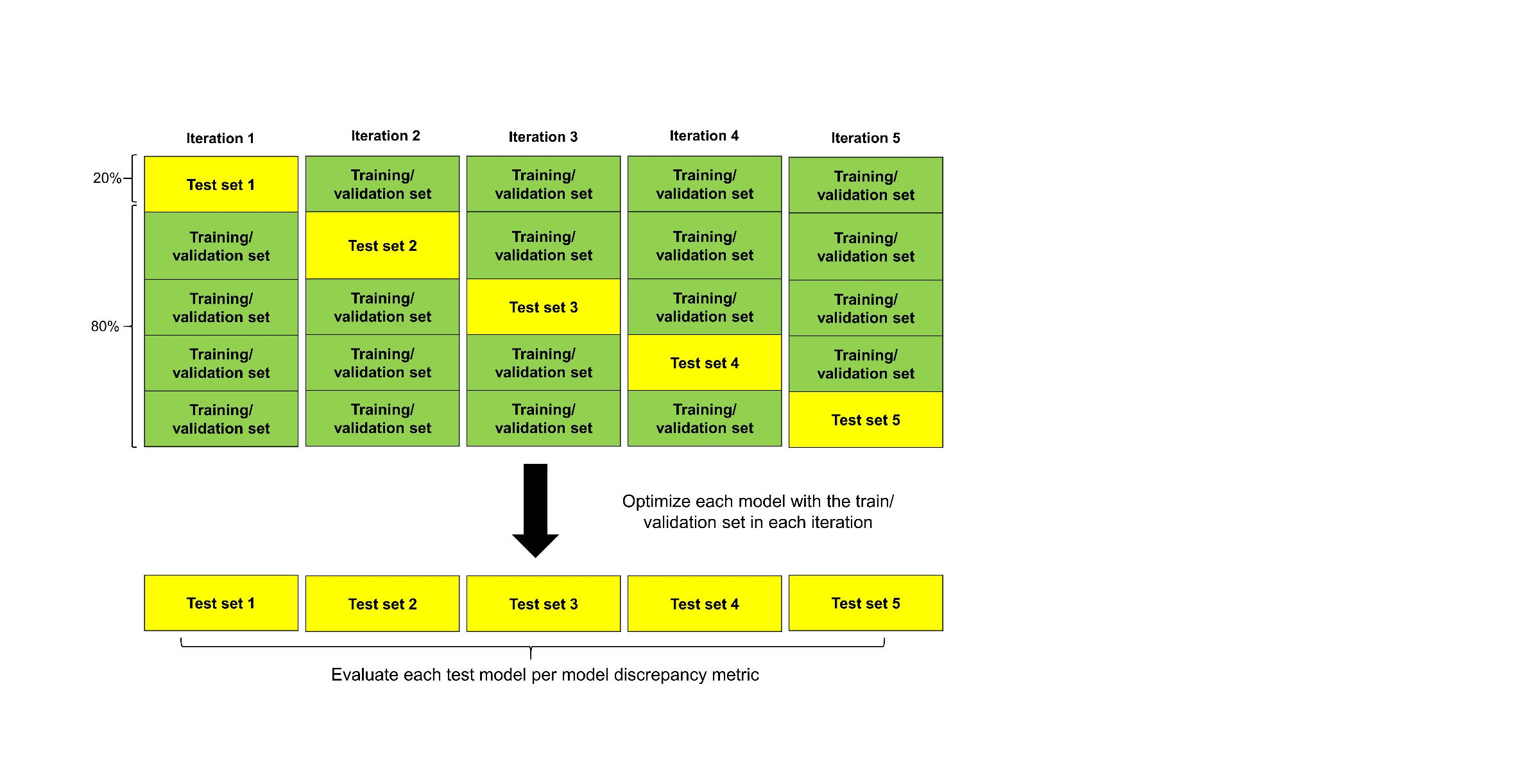


1. Nested 5-fold cross-validation for XGBoost and SVM


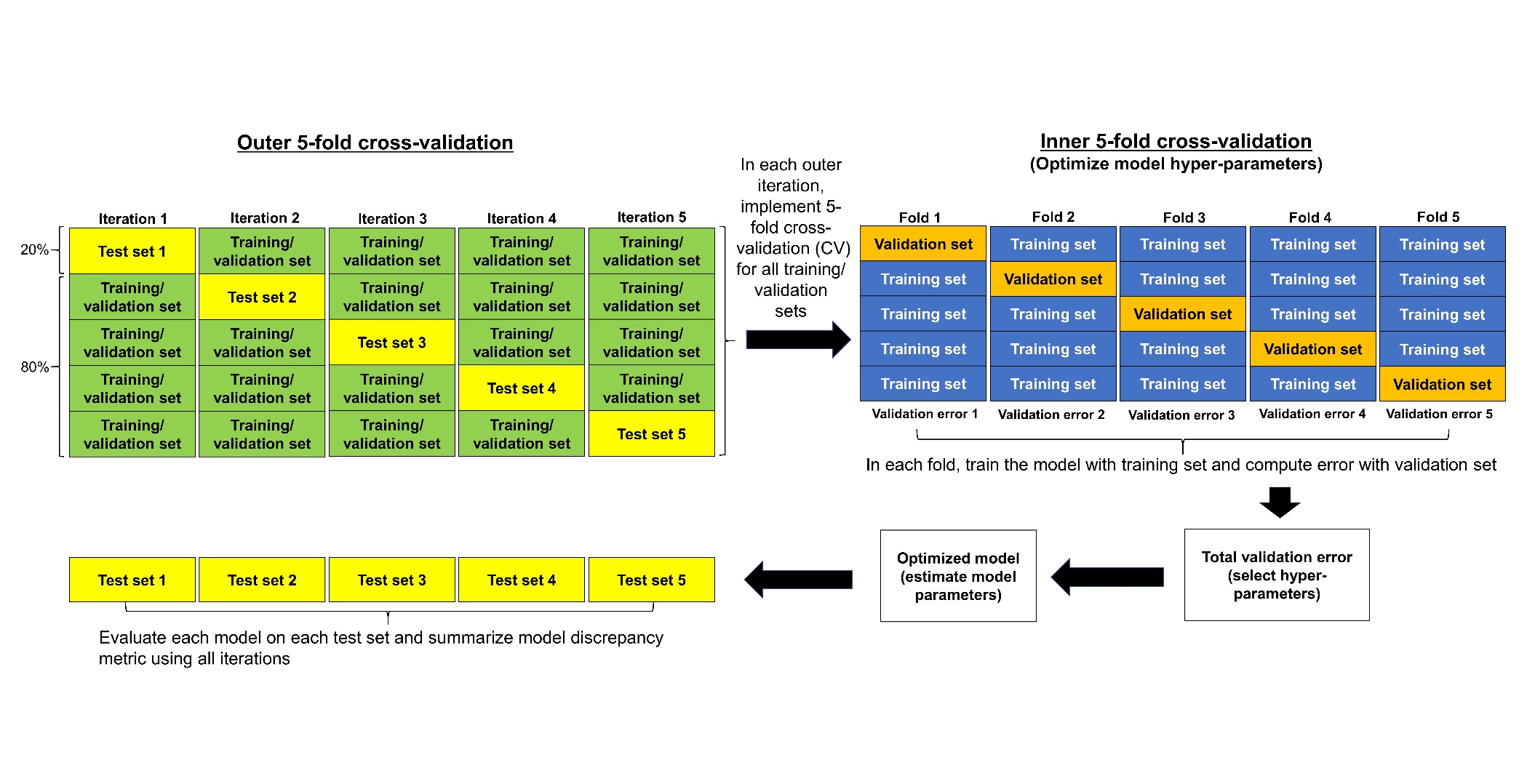


Abbreviations:

BERT, Bidirectional Encoder Representations from Transformers; SVM, Support Vector Machine; XGBoost, eXtreme Gradient Boosting
