# Supplementary material for: Natural Language Processing and Machine Learning Methods to Characterize Unstructured Patient-Reported Outcomes: Validation Study
Source: J Med Internet Res. 2021 Nov 3;23(11):e26777. doi: 10.2196/26777 (PMC8600437; doi:10.2196/26777)
Supplement: Multimedia Appendix 12 [file jmir_v23i11e26777_app12.docx]

Table S8: Frequency of attributes in pain interference and fatigue domains labeled by content experts

| Domains | Attributes | Expert-labeled symptom attributes, N (%) | | |
| --- | --- | --- | --- | --- |
|  |  | Survivors | Caregivers | Total |
| Pain interference | Total | 255 (65.2) | 136 (34.8) | 391 |
|  | Physical | 52 (73.2) | 19 (26.8) | 71 |
|  | Cognitive | 37 (77.1) | 11 (22.9) | 48 |
|  | Social | 27 (64.3) | 15 (35.7) | 42 |
|  | Other | 139 (60.4) | 91 (39.6) | 230 |
| Fatigue | Total | 275 (65.0) | 148 (35.0) | 423 |
|  | Physical | 49 (59.8) | 33 (40.2) | 82 |
|  | Cognitive | 53 (75.7) | 17 (24.3) | 70 |
|  | Social | 34 (75.6) | 11 (24.4) | 45 |
|  | Other | 139 (61.5) | 87 (38.5) | 226 |
