# Supplementary material for: Natural Language Processing and Machine Learning Methods to Characterize Unstructured Patient-Reported Outcomes: Validation Study
Source: J Med Internet Res. 2021 Nov 3;23(11):e26777. doi: 10.2196/26777 (PMC8600437; doi:10.2196/26777)
Supplement: Multimedia Appendix 13 [file jmir_v23i11e26777_app13.docx]

Table S9: The changes of the area under the receiver operating characteristic curve and precision-recall curve among different NLP/ML models (survivors and caregivers)

| Domains | Attributes | Changes in AUROCC (95% CI) | | | Changes in AUPRC (95% CI) | | |
| --- | --- | --- | --- | --- | --- | --- | --- |
|  |  | Word2vec/SVM vs. Word2vec/XGBoost | BERT vs. Word2vec/SVM | BERT vs. Word2vec/XGBoost | Word2vec/SVM vs. Word2vec/XGBoost | BERT vs. Word2vec/SVM | BERT vs. Word2vec/XGBoost |
| Pain inter-ference | Physical | 0.038  (-0.016, 0.095) | 0.007  (-0.040, 0.055) | 0.045  (-0.011, 0.104) | 0.070  (-0.031, 0.172) | 0.054  (-0.057, 0.160) | 0.124  (0.003, 0.252) |
|  | Cognitive | 0.072  (0.014, 0.128) | 0.023  (-0.027, 0.070) | 0.095  (0.054, 0.147) | 0.135  (-0.015, 0.283) | 0.209  (0.091, 0.329) | 0.344  (0.211, 0.459) |
|  | Social | 0.018  (-0.063, 0.089) | 0.053  (-0.039, 0.140) | 0.071  (-0.011, 0.150) | 0.005  (-0.105, 0.096) | 0.257  (0.102, 0.390) | 0.262  (0.073, 0.406) |
| Fatigue | Physical | 0  (-0.059, 0.061) | 0.049  (-0.021, 0.126) | 0.049  (-0.024, 0.117) | -0.086  (-0.173, 0.014) | 0.162  (0.052, 0.263) | 0.076  (-0.027, 0.175) |
|  | Cognitive | 0.063  (0.019, 0.100) | 0.031  (-0.005, 0.066) | 0.057  (0.040, 0.135) | 0.071  (-0.028, 0.170) | 0.125  (0.048, 0.200) | 0.196  (0.096, 0.308) |
|  | Social | 0.037  (-0.028, 0.096) | -0.021  (-0.107, 0.055) | 0.016  (-0.080, 0.107) | 0.063  (-0.081, 0.213) | 0.168  (0.058, 0.265) | 0.231  (0.055, 0.381) |

Abbreviations:

AUPRC, area under precision-recall curve; AUROCC, area under the receiver operating characteristic curve; BERT, Bidirectional Encoder Representations from Transformers; CI, confidence interval; ML, machine learning; NLP, natural language processing; SVM, Support Vector Machine; XGBoost, eXtreme Gradient Boosting
