# Supplementary material for: Natural Language Processing and Machine Learning Methods to Characterize Unstructured Patient-Reported Outcomes: Validation Study
Source: J Med Internet Res. 2021 Nov 3;23(11):e26777. doi: 10.2196/26777 (PMC8600437; doi:10.2196/26777)
Supplement: Multimedia Appendix 14 [file jmir_v23i11e26777_app14.docx]

Figure S5: Precision-recall curves for pain interference and fatigue domains by

three symptom attributes (survivors and caregivers)

| Pain interference domain –  cognitive attribute | Fatigue domain –  cognitive attribute |
| --- | --- |
| 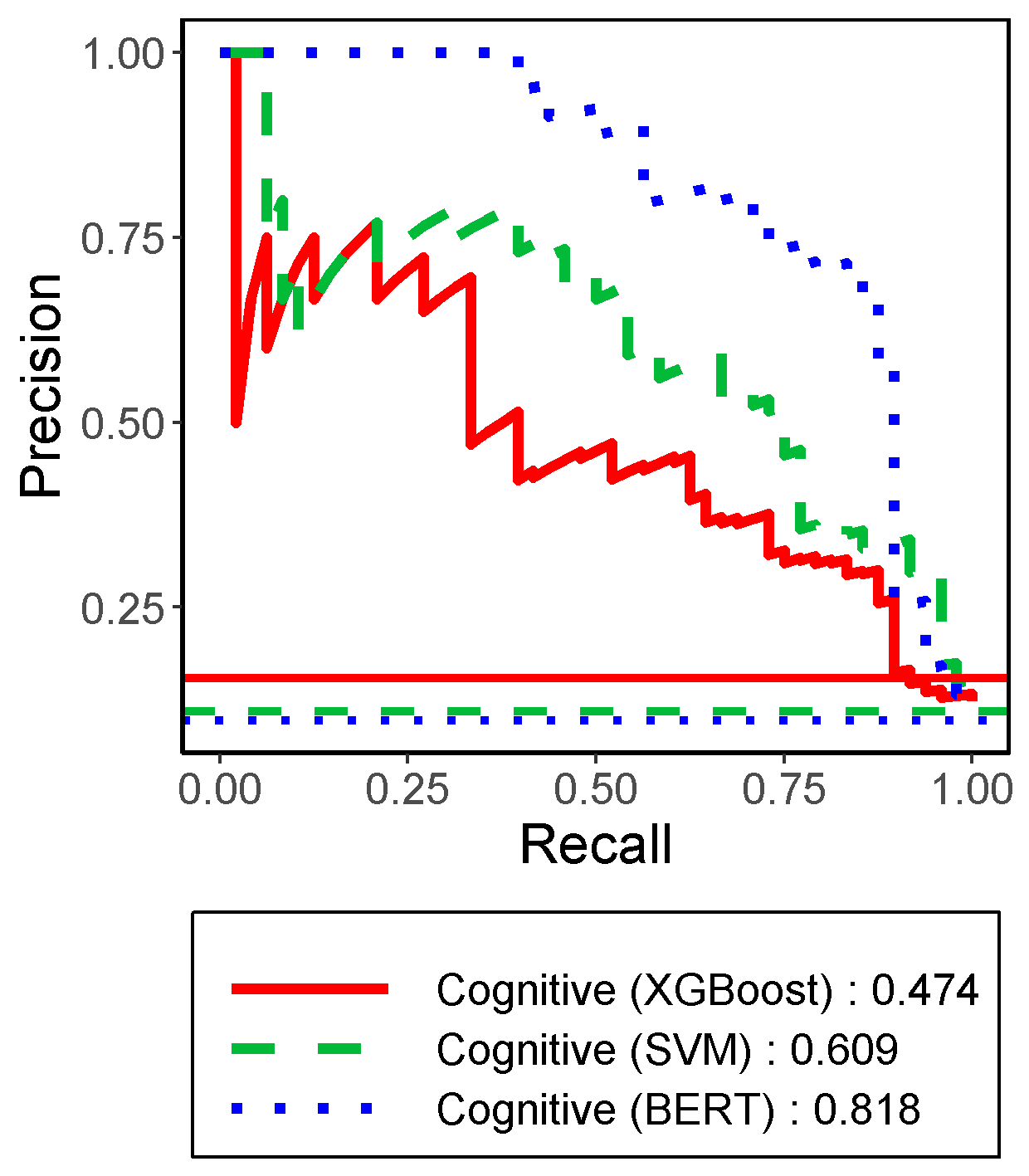 | 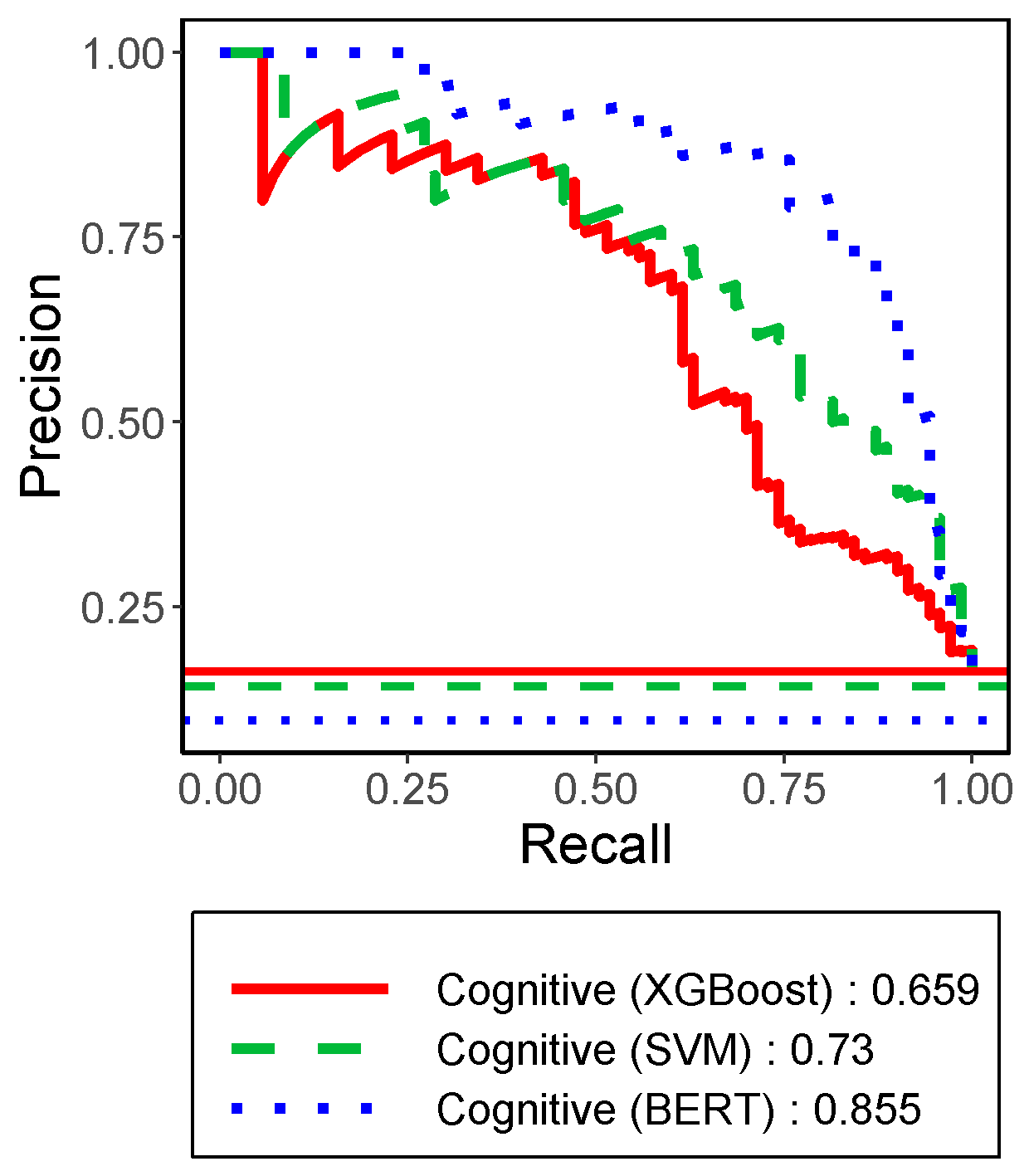 |
|  |  |
| Pain interference domain –  physical attribute | Fatigue domain –  physical attribute |
| 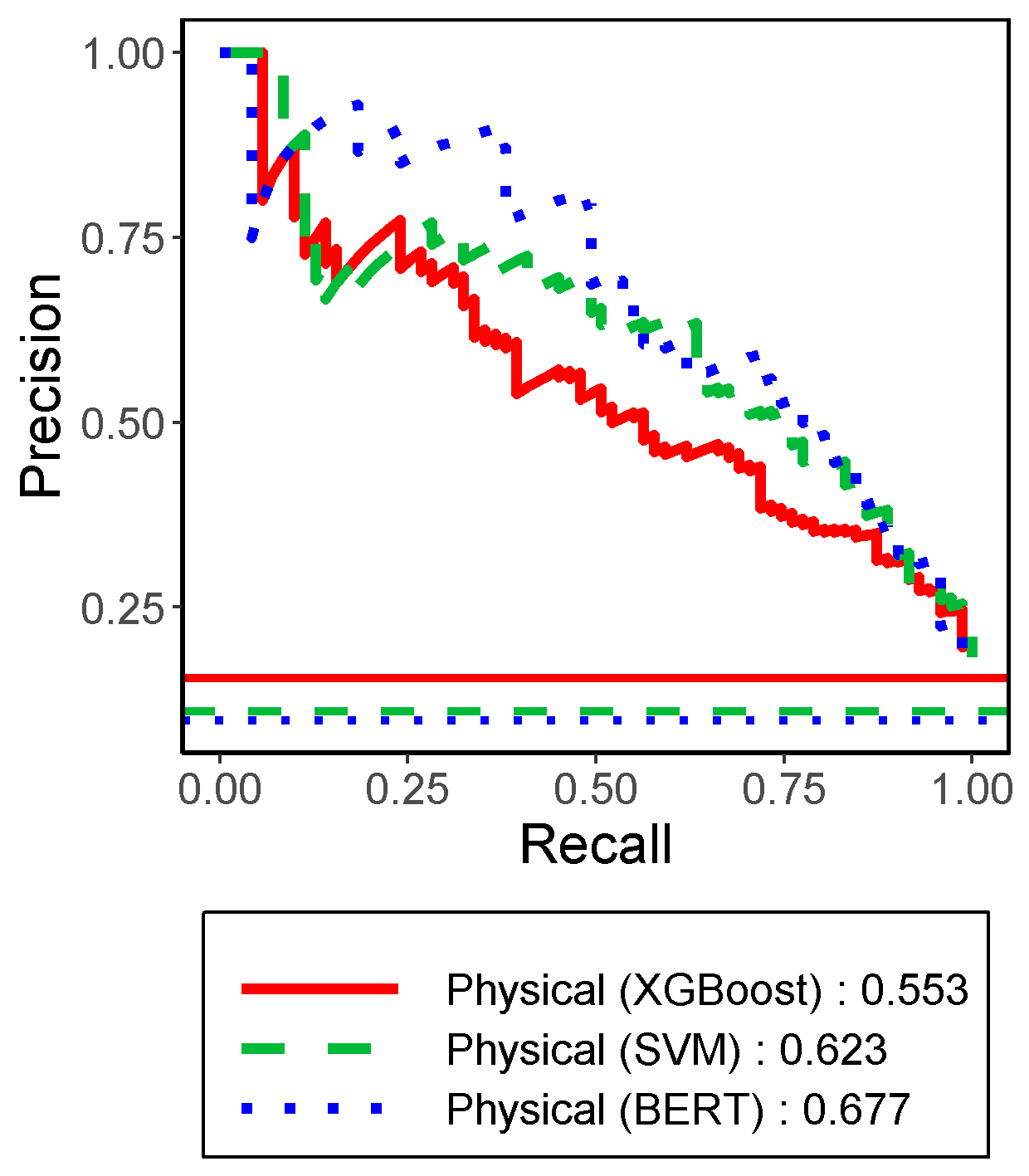 | 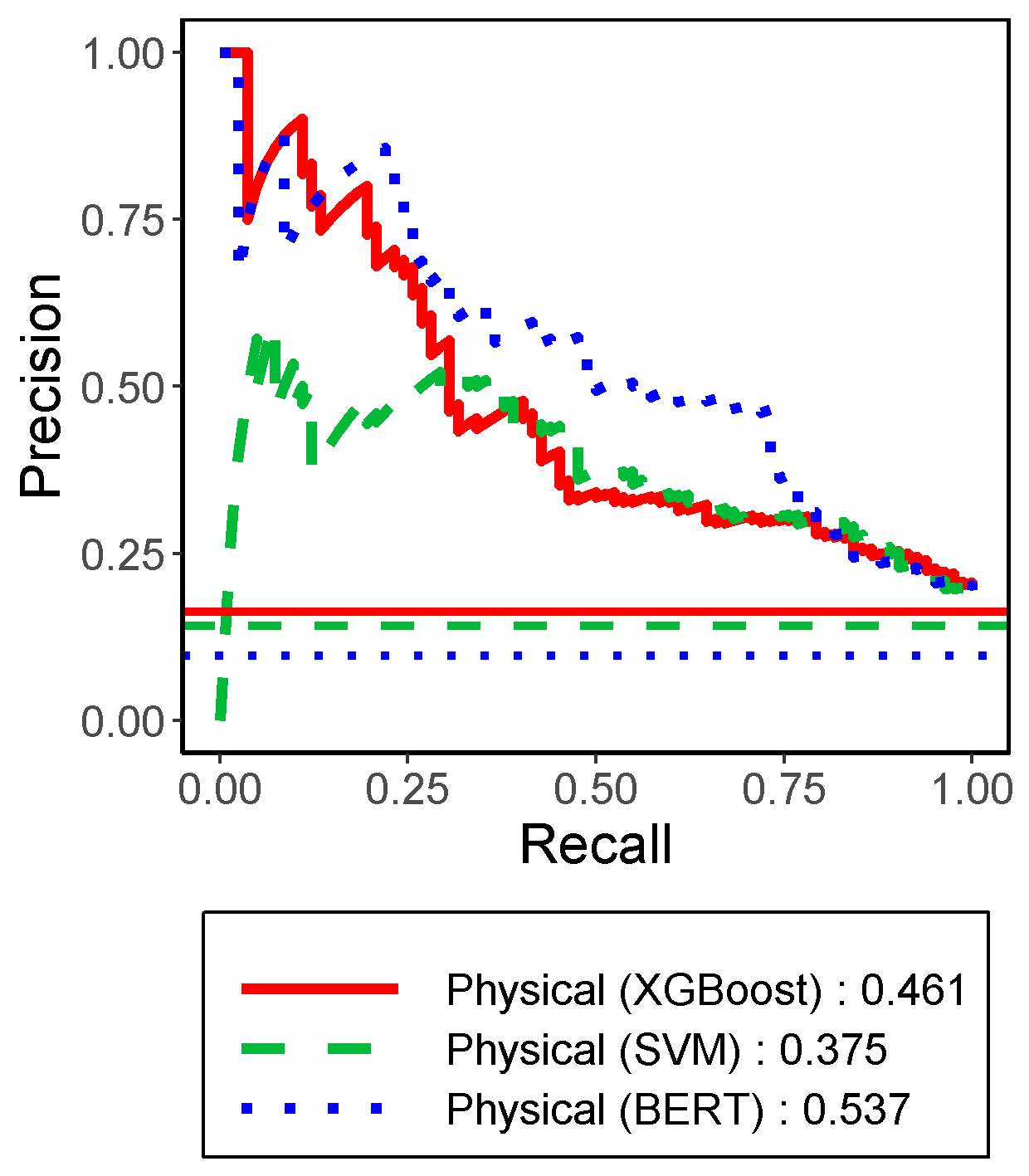 |
|  |  |

| Pain interference domain –  social attribute | Fatigue domain –  social attribute |
| --- | --- |
| 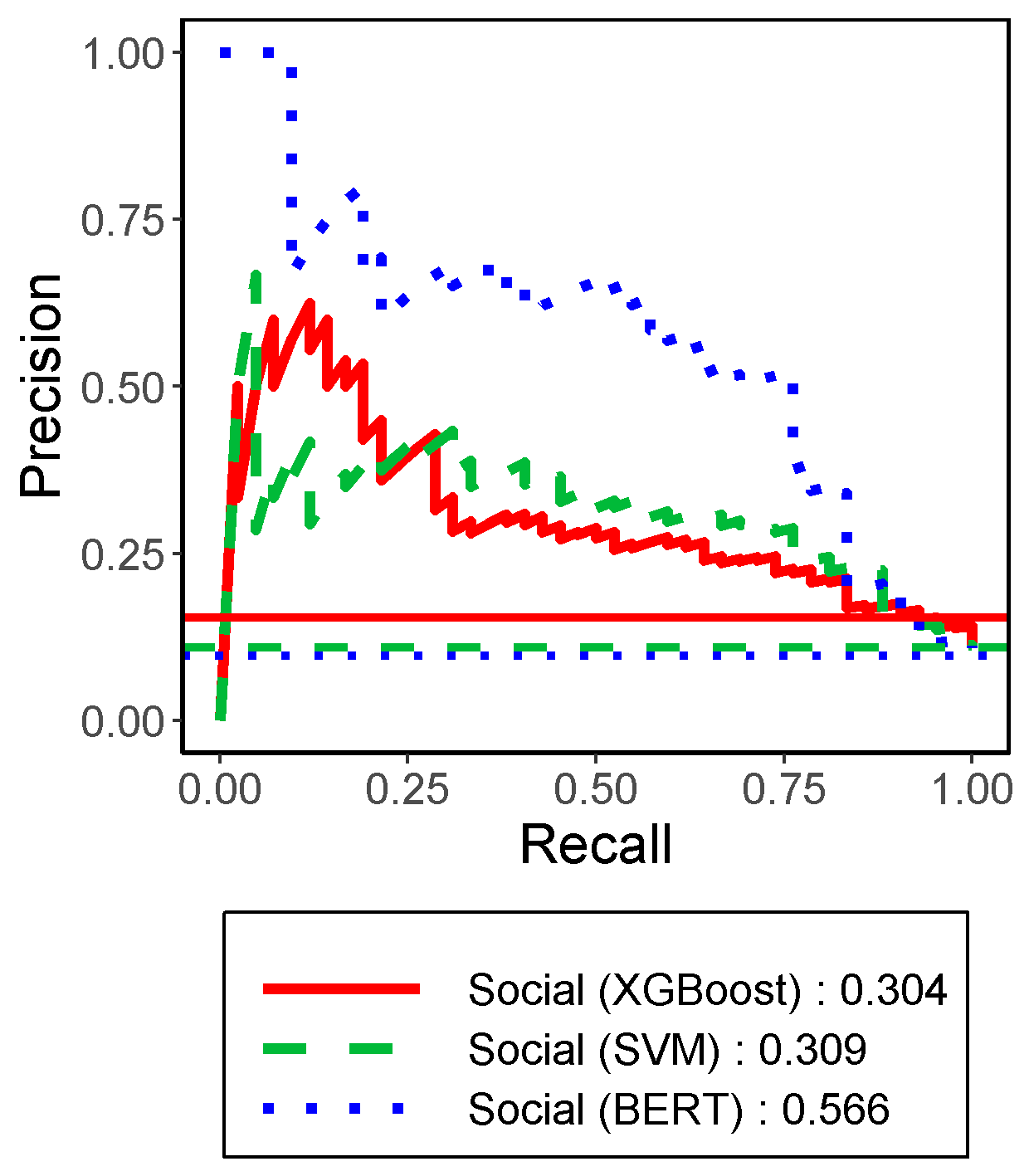 | 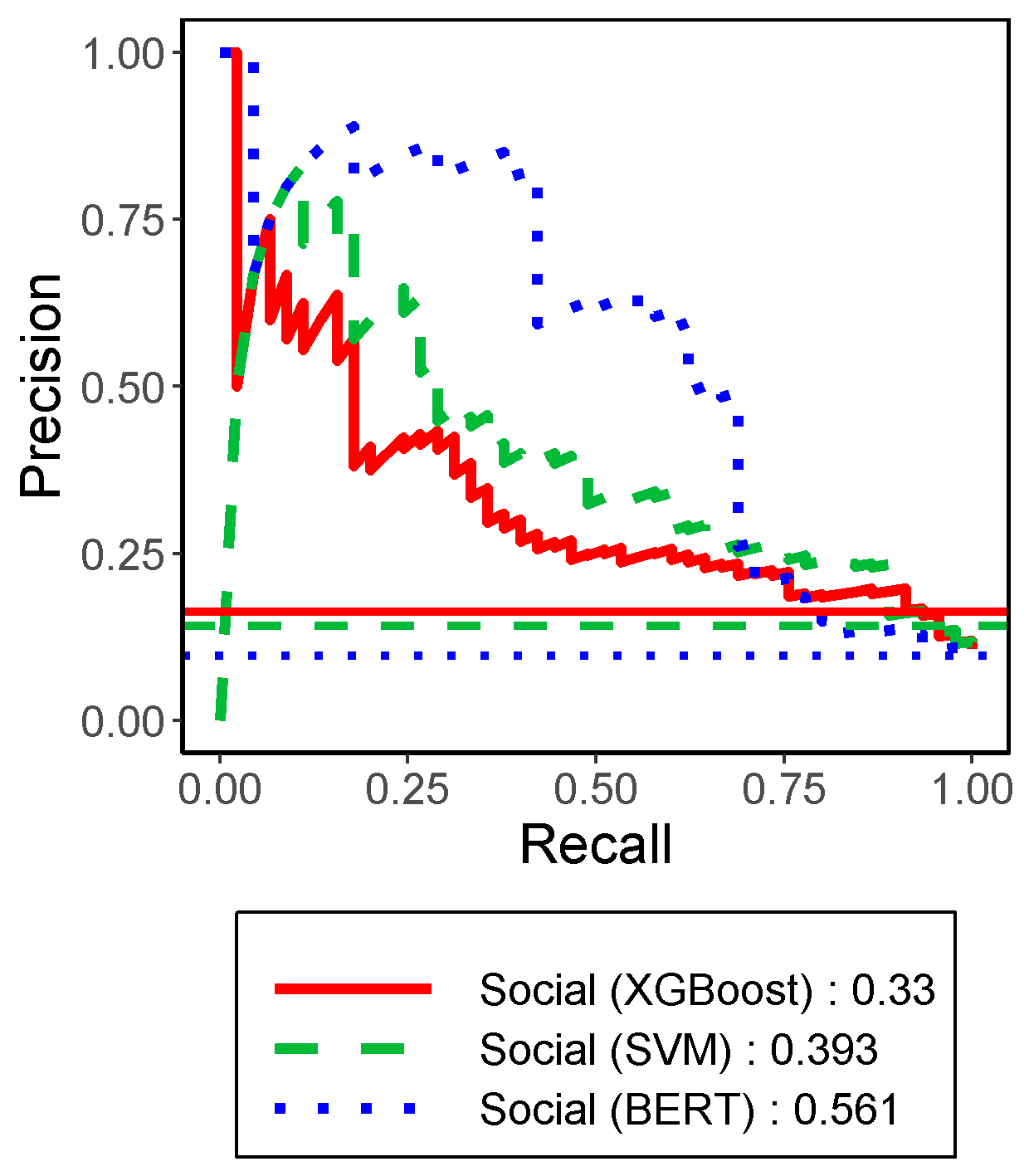 |
|  |  |

Abbreviations:

XGBoost, eXtreme Gradient Boosting; SVM, Support Vector Machine; BERT, Bidirectional Encoder Representations from Transformers
