# Supplementary material for: Natural Language Processing and Machine Learning Methods to Characterize Unstructured Patient-Reported Outcomes: Validation Study
Source: J Med Internet Res. 2021 Nov 3;23(11):e26777. doi: 10.2196/26777 (PMC8600437; doi:10.2196/26777)
Supplement: Multimedia Appendix 15 [file jmir_v23i11e26777_app15.docx]

Table S10: Performance of NLP/ML models for pain interference domain by three symptom attributes (survivors only)

| Attributes | Models | Precision  (95% CI) | Sensitivity  (95% CI) | Specificity  (95% CI) | Accuracy  (95% CI) | F1  (95% CI) | AUROCC  (95% CI) | AUPRC  (95% CI) |
| --- | --- | --- | --- | --- | --- | --- | --- | --- |
| Physical | BERT | 0.595  (0.441, 0.750) | 0.423  (0.291, 0.562) | 0.926  (0.888, 0.961) | 0.824  (0.773, 0.867) | 0.494  (0.359, 0.614) | 0.783  (0.703, 0.873) | 0.574  (0.415, 0.749) |
|  | Word2vec/SVM | 0.550  (0.333, 0.773) | 0.212  (0.105, 0.327) | 0.956  (0.925, 0.981) | 0.804  (0.753, 0.851) | 0.306  (0.161, 0.439) | 0.788  (0.701, 0.894) | 0.517  (0.392, 0.637) |
|  | Word2vec/XGBoost | 0.500  (0.292, 0.708) | 0.231  (0.122, 0.349) | 0.941  (0.905, 0.971) | 0.796  (0.745, 0.843) | 0.316  (0.174, 0.444) | 0.734  (0.662, 0.811) | 0.461  (0.320, 0.601) |
| Cognitive | BERT | 0.889  (0.741, 1.000) | 0.649  (0.500, 0.784) | 0.986  (0.968, 1.000) | 0.937  (0.906, 0.961) | 0.750  (0.613, 0.846) | 0.896  (0.826, 0.962) | 0.809  (0.719, 0.956) |
|  | Word2vec/SVM | 0.708  (0.524, 0.885) | 0.459  (0.300, 0.611) | 0.968  (0.943, 0.991) | 0.894  (0.855, 0.925) | 0.557  (0.400, 0.689) | 0.889  (0.835, 0.957) | 0.665  (0.522, 0.846) |
|  | Word2vec/XGBoost | 1.000  (1.000, 1.000) | 0.243  (0.107, 0.395) | 1.000  (1.000, 1.000) | 0.890  (0.851, 0.925) | 0.391  (0.195, 0.566) | 0.822  (0.745, 0.910) | 0.559  (0.438, 0.708) |
| Social | BERT | 0.500  (0.143, 0.833) | 0.148  (0.033, 0.280) | 0.982  (0.965, 0.996) | 0.894  (0.851, 0.929) | 0.229  (0.065, 0.391) | 0.800  (0.689, 0.920) | 0.397  (0.182, 0.596) |
|  | Word2vec/SVM | 0  (0, 0) | 0  (0, 0) | 0.996  (0.987, 1.000) | 0.890  (0.851, 0.929) | NA | 0.773  (0.712, 0.855) | 0.215  (0.062, 0.304) |
|  | Word2vec/XGBoost | 0.250  (0, 1.000) | 0.037  (0, 0.125) | 0.987  (0.970, 1.000) | 0.886  (0.843, 0.925) | 0.065  (0.050, 0.207) | 0.724  (0.628, 0.829) | 0.241  (0.105, 0.358) |

Abbreviations:

AUPRC, area under precision-recall curve; AUROCC, area under the receiver operating characteristic curve; BERT, Bidirectional Encoder Representations from Transformers; CI, confidence interval; ML, machine learning; NLP, natural language processing; SVM, Support Vector Machine; XGBoost, eXtreme Gradient Boosting
