# Supplementary material for: Natural Language Processing and Machine Learning Methods to Characterize Unstructured Patient-Reported Outcomes: Validation Study
Source: J Med Internet Res. 2021 Nov 3;23(11):e26777. doi: 10.2196/26777 (PMC8600437; doi:10.2196/26777)
Supplement: Multimedia Appendix 16 [file jmir_v23i11e26777_app16.docx]

Table S11: Performance of NLP/ML models for fatigue domain by three symptom attributes (survivors only)

| Attributes | Models | Precision  (95% CI) | Sensitivity  (95% CI) | Specificity  (95% CI) | Accuracy  (95% CI) | F1  (95% CI) | AUROCC  (95% CI) | AUPRC  (95% CI) |
| --- | --- | --- | --- | --- | --- | --- | --- | --- |
| Physical | BERT | 0.512  (0.361, 0.660) | 0.449  (0.308, 0.596) | 0.907  (0.870, 0.941) | 0.825  (0.778, 0.873) | 0.478  (0.337, 0.598) | 0.820  (0.760, 0.893) | 0.556  (0.403, 0.675) |
|  | Word2vec/SVM | 0.571  (0.278, 0.834) | 0.163  (0.061, 0.278) | 0.973  (0.951, 0.991) | 0.829  (0.785, 0.873) | 0.254  (0.100, 0.400) | 0.759  (0.679, 0.854) | 0.439  (0.310, 0.557) |
|  | Word2vec/XGBoost | 0.593  (0.400, 0.786) | 0.327  (0.196, 0.471) | 0.951  (0.920, 0.978) | 0.840  (0.793, 0.884) | 0.421  (0.271, 0.553) | 0.761  (0.685, 0.839) | 0.468  (0.312, 0.627) |
| Cognitive | BERT | 0.808  (0.698, 0.913) | 0.792  (0.679, 0.900) | 0.955  (0.925, 0.981) | 0.924  (0.891, 0.953) | 0.800  (0.707, 0.880) | 0.923  (0.877, 0.981) | 0.841  (0.757, 0.946) |
|  | Word2vec/SVM | 0.828  (0.676, 0.960) | 0.453  (0.320, 0.600) | 0.977  (0.956, 0.995) | 0.876  (0.836, 0.913) | 0.585  (0.450, 0.706) | 0.905  (0.866, 0.967) | 0.754  (0.643, 0.896) |
|  | Word2vec/XGBoost | 0.625  (0.452, 0.792) | 0.377  (0.250, 0.511) | 0.946  (0.913, 0.973) | 0.836  (0.793, 0.880) | 0.471  (0.333, 0.600) | 0.814  (0.747, 0.868) | 0.597  (0.477, 0.733) |
| Social | BERT | 0.706  (0.480, 0.929) | 0.353  (0.200, 0.514) | 0.979  (0.960, 0.996) | 0.902  (0.865, 0.935) | 0.471  (0.293, 0.621) | 0.805  (0.708, 0.907) | 0.539  (0.426, 0.703) |
|  | Word2vec/SVM | 0.400  (0.100, 0.714) | 0.118  (0.026, 0.229) | 0.975  (0.953, 0.992) | 0.869  (0.822, 0.909) | 0.182  (0.049, 0.333) | 0.801  (0.713, 0.874) | 0.339  (0.155, 0.479) |
|  | Word2vec/XGBoost | 0.778  (0.429, 1.000) | 0.206  (0.062, 0.345) | 0.992  (0.979, 1.000) | 0.895  (0.855, 0.927) | 0.326  (0.118, 0.500) | 0.737  (0.657, 0.822) | 0.373  (0.197, 0.571) |

Abbreviations:

AUPRC, area under precision-recall curve; AUROCC, area under the receiver operating characteristic curve; BERT, Bidirectional Encoder Representations from Transformers; CI, confidence interval; ML, machine learning; NLP, natural language processing; SVM, Support Vector Machine; XGBoost, eXtreme Gradient Boosting
